# Efficacy and safety of biologics for hidradenitis suppurativa: A network meta‐analysis of phase III trials

**DOI:** 10.1111/jdv.20617

**Published:** 2025-03-10

**Authors:** Laura Calabrese, Alessandra Cartocci, Pietro Rubegni, Lars E. French, Benjamin Kendziora

**Affiliations:** ^1^ Dermatology Unit, Department of Medical, Surgical and Neurological Sciences University of Siena Siena Italy; ^2^ Institute of Dermatology Catholic University of the Sacred Heart Rome Italy; ^3^ Department of Dermatology and Allergy University Hospital, LMU Munich Germany; ^4^ Department of Dermatology, Thalkirchner Street Hospital Munich Municipial Hospital Group Munich Germany; ^5^ Dr. Phillip Frost Department of Dermatology and Cutaneous Surgery, Miller School of Medicine University of Miami Miami Florida USA; ^6^ Institute for Health Services Research in Dermatology and Nursing (IVDP), University Medical Center Hamburg‐Eppendorf University of Hamburg Hamburg Germany

## Abstract

**Background:**

Phase III clinical trials are designed to evaluate the therapeutic effect of drugs and their superiority over other treatment methods, but biologics for hidradenitis suppurativa (HS) have not been compared head‐to‐head in phase III studies.

**Objectives:**

To evaluate the relative efficacy and safety of biologics for HS in a network meta‐analysis including available data from phase III trials.

**Methods:**

MEDLINE and Embase were searched for phase III trials investigating the efficacy and/or safety of at least one biologic for moderate‐to‐severe HS. The odds ratios for reaching an HS Clinical Response 50 (HiSCR50) and for the occurrence of adverse events after 12–16 weeks were compared between treatments.

**Results:**

PIONEER I and II (adalimumab 40 mg weekly vs. placebo), SUNSHINE and SUNRISE (secukinumab 300 mg every 2 vs. 4 weeks vs. placebo) as well as BE HEARD I and II (bimekizumab 320 mg every 2 vs. 4 weeks vs. placebo) with 2731 patients were included. Adalimumab weekly was ranked most effective in reaching a HiSCR50 with significant superiority over secukinumab every 2 weeks (OR = 1.74; 95% confidence interval [CI]: 1.11–2.73) and 4 weeks (OR = 1.72; 95% CI: 1.09–2.7) and insignificant superiority over bimekizumab every 2 weeks (OR = 1.23; 95% CI: 0.74–2.06) and 4 weeks (OR = 1.25; 95% CI: 0.73–2.14). Adalimumab showed the fewest adverse events with significant superiority over bimekizumab every 2 weeks (OR = 0.52; 95% CI: 0.32–0.86) and insignificant superiority over bimekizumab every 4 weeks (OR = 0.79; 95% CI: 0.47–1.33) and secukinumab every 2 weeks (OR = 0.69; 95% CI: 0.45–1.07) and 4 weeks (OR = 0.71; 95% CI: 0.46–1.1).

**Conclusions:**

Among currently approved biologic agents for moderate‐to‐severe HS, adalimumab demonstrated the highest efficacy and safety in the first 12–16 weeks of treatment.

## Graphical abstract

Data from 2731 patients with hidradenitis suppurativa (HS) treated with biologics for 12–16 weeks in phase III randomized controlled trials (RCT) were included in a network meta‐analysis (NMA). Adalimumab showed the highest efficacy in achieving a clinical HS response 50 (HiSCR50) and the lowest incidence of adverse events (AE). The *P*‐Score is a statistical metric that provides a measure of how likely a treatment is the most effective or safest option among analyzed treatments while accounting for uncertainty.

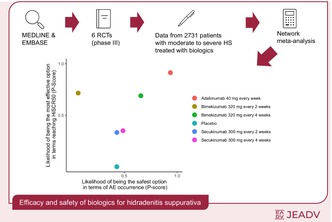

**Why was the study undertaken?**

- To identify the most effective and safest biologics for moderate‐to‐severe hidradenitis suppurativa (HS).

**What does the study add?**

- Recently published phase III data on bimekizumab were included in this network meta‐analysis and compared with phase III data on adalimumab and secukinumab, focusing on the first 12–16 weeks of HS treatment.
- Adalimumab weekly was ranked the most effective in reaching an HS Clinical Response 50 with significant superiority over secukinumab every 2 weeks and every 4 weeks and insignificant superiority over bimekizumab every 2 weeks and every 4 weeks.
- Adalimumab showed the fewest adverse events, with significant superiority over bimekizumab every 2 weeks and insignificant superiority over bimekizumab every 4 weeks and secukinumab every 2 weeks and every 4 weeks.

**What are the implications of this study for disease understanding and/or clinical care?**

- Among currently approved biologic agents for moderate‐to‐severe HS, adalimumab demonstrated the highest efficacy and safety in the first 12–16 weeks of treatment.

## INTRODUCTION

Hidradenitis suppurativa (HS) is a chronic inflammatory skin disease affecting approximately 1% of the general population.^1^ Clinical manifestations of HS, comprising painful inflammatory nodules, abscesses, fistulas, and scars located mainly in intertriginous areas, considerably affect patients' quality of life.^2^, ^3^

Treatment options are currently adapted to disease severity and include pharmacological and/or surgical intervention, combined with supportive care and lifestyle modifications.^4^, ^5^, ^6^, ^7^ The tumour necrosis factor (TNF) α antagonist adalimumab was the first biologic approved for the therapy of moderate‐to‐severe HS. Results from phase III clinical trials demonstrated a clinically meaningful Hidradenitis Suppurativa Clinical Response 50 (HiSCR50) in 41.8 and 58.9% of patients receiving Adalimumab 40 mg weekly compared to 26.0 and 27.6% of patients receiving placebo, respectively.^8^

Secukinumab, a human monoclonal antibody (mAb) targeting IL‐17A, was the first agent in this class to be approved for the treatment of moderate to severe HS at a dose of 300 mg weekly for 5 weeks and once monthly thereafter. More recently, bimekizumab, a humanized mAb that simultaneously inhibits both IL‐17A and IL‐17F, was approved with a dosing of 320 mg every 2 weeks up to week 16 and every 4 weeks thereafter.^9^, ^10^, ^11^

To date, no randomized head‐to‐head trials between adalimumab, secukinumab, and bimekizumab have been conducted in HS; therefore, educating clinical decision‐making still remains challenging.

Network meta‐analyses (NMAs) are a powerful tool for the best possible comparative analysis of available evidence concerning the efficacy, safety, and ultimate therapeutic value of available treatments that have not been directly compared in a randomized controlled trial (RCT).

Although NMAs comparing the outcomes of systemic therapies for HS have been published,^12^, ^13^, ^14^ no specific comparisons were made between biologic agents that have completed phase III RCTs, and none of the existing NMAs included data from the recently concluded phase III trial of bimekizumab. The rationale behind the exclusive inclusion of phase III studies in this NMA was that the quality of an NMA increases with the similarity of the studies included^15^ and phase III studies are quite standardized in terms of methodology.

The aim of this study was to investigate the comparative efficacy and safety of biologic agents for the treatment of moderate‐to‐severe HS by including data from phase III RCTs.

## MATERIALS AND METHODS

### Eligibility criteria

#### Population

Studies including adults (aged ≥18 years) with moderate‐to‐severe HS were eligible; more specifically, studies that included only or also adults with moderate‐to‐severe HS.

#### Study design, interventions and comparators

Phase III placebo‐controlled and head‐to‐head RCTs investigating the efficacy and/or safety of at least one biologic agent (adulimumab, secukinumab and bimekizumab). Comparators could be an active comparator or placebo. Interventions and comparators could be administered in monotherapy or in association with systemic antibiotics or topical antiseptics (combination therapy). Non‐phase III trials, studies with a non‐randomized design, studies with a run‐in period with a systemic therapy, meeting abstracts, and trials with trial registration entry but early termination were excluded.

#### Outcomes

The primary efficacy outcome was the Hidradenitis Suppurativa Clinical Response 50 (HiSCR50) after 12–16 weeks of treatment. HiSCR50 is defined as a ≥50% decrease in abscess and inflammatory nodules count with no increase in the number of abscesses and/or in the number of draining fistulae compared with baseline.^16^ The primary safety outcome was the occurrence of adverse events (AEs) during 12–16 weeks of treatment. The occurrence of serious adverse events (SAEs) during 12–16 weeks of treatment was a secondary outcome. The 30% reduction in pain on a numerical rating scale after 12–16 weeks was another planned secondary outcome that could not be analysed due to insufficient data.

### Search strategy, screening of references and data extraction

Eligible trials were searched via Ovid in MEDLINE and Embase until 18 June 2023. The search term used in Ovid can be found in Table S1 in Appendix A. From a search on clinicaltrials.gov, we knew that the phase III trials BE HEARD I and BE HEARD II^10^ were ongoing at the date of our search. Upon request, the sponsor kindly sent us a brief summary of the data. However, in order to obtain all the necessary data for inclusion in the NMA, we decided to await the full publication of the results. To be on the safe side, the reference lists of included studies were reviewed for studies that we might have missed. Screening of references and data extraction was performed independently in duplicate; details can be found in Appendix A.

### Data analysis

#### Measure of effect

The odds ratio (OR) was applied as an effect measure.

#### Network meta‐analysis

To allow head‐to‐head comparisons for all treatments, a frequentist random effects NMA model was built for each outcome if sufficient data were available. The reference group was placebo. A forest plot was created to display the effect size of each treatment relative to placebo. All head‐to‐head comparisons were summarized in a league table. Moreover, the treatments were ranked according to *P*‐scores.^17^

#### Quality of evidence

The risk of bias within included studies was evaluated using the revised Cochrane Risk of Bias Tool 2.^18^ Publication bias across studies was assessed by Egger's regression test. The GRADE Working Group approach was used to rate the certainty in all treatment effect estimates relative to placebo.^19^ Further details on the evaluation of the quality of evidence can be found in Appendix A.

#### Sensitivity analyses

Results of the primary analyses of the included trials were used for the primary analysis of this NMA. Patients receiving systemic antibiotics as rescue for HS were counted as non‐responders for the outcome HiSCR50 in the primary analyses of PIONEER I and II, SUNSHINE and SUNRISE, while patients who took any systemic antibiotic, not only as rescue for HS, were treated as non‐responders in the primary analysis of BE HEARD I and II. The latter is very stringent and may underestimate the efficacy of bimekizumab. Therefore, we performed a sensitivity analysis in which patients included in BE HEARD I and II who were treated with a systemic antibiotic were only counted as non‐responders if the systemic antibiotic was used as rescue therapy for HS, similar to the designs of PIONEER I and II, SUNSHINE and SUNRISE.

Worsening of HS was counted as an AE in all included studies. Worsening of HS is likely connected to treatment performance. We performed a sensitivity analysis on the occurrence of AEs without counting worsening of HS as an AE. This allows for a comparison of the occurrence of AEs between treatments more independently of the efficacy. The definition of an AE in clinical trials is standardized and well established as any untoward medical occurrence temporally associated with the treatment, regardless of its actual causality.^20^ Therefore, by including HS flares in the primary analysis, we maintained consistency with this definition. Conversely, excluding flares represented a narrower focus and was conducted as a sensitivity analysis to evaluate the robustness of our results.

#### Performance of the statistical analysis and software

Details can be found in Appendix A.

### Protocol

The protocol for this NMA was registered and published in PROSPERO (CRD42023405391).

## RESULTS

### Included studies and patients

**FIGURE 1:**
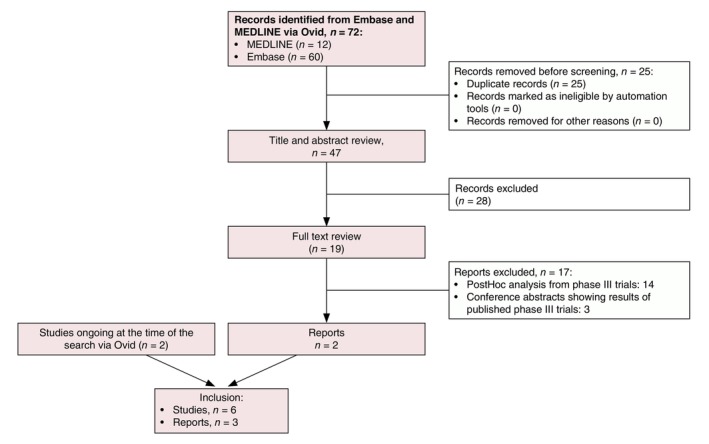
Screening of references. Six phase III trials were included in total. Four trials were identified by the search in MEDLINE and Embase via Ovid. Two trials were ongoing at the time of the search via Ovid and were included after publication.

**FIGURE 2:**
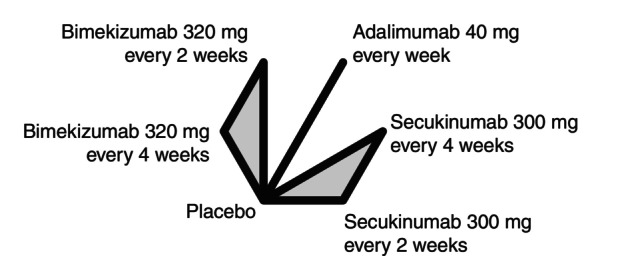
Network graph with included treatments. Lines represent direct comparisons performed by the included phase III trials. Grey triangles depict multi‐arm studies.

The search in MEDLINE and Embase revealed 72 references. Following the title, abstract and full‐text review, four trials were considered eligible: PIONEER I, PIONEER II,^8^ SUNSHINE and SUNRISE.^9^ As mentioned above, we knew that BE HEARD I and BE HEARD II^10^ were ongoing at the date of our search and included these trials after publication (Figure 1). No study falsely appeared to meet the inclusion criteria and was subsequently excluded. All excluded references with the reasons for exclusion are listed in Table A2. The study designs and the usage of concomitant antibiotics in the included studies are summarized in Appendix A. Figure 2 shows a network graph, which depicts the treatment comparisons performed with the included trials and how these direct comparisons were integrated into a network, allowing direct and indirect treatment comparisons. In total, 2731 patients were included in this NMA. Baseline patient characteristics are summarized in Table 1. The baseline characteristics for each individual study are listed in Table A3.

**TABLE 1:** Main characteristics of included patients.

Number of studies, *n*	6
Randomized patients, *n*	2731
Age, mean years (95% CI)	36.0 (35.5; 36.5)
BMI, mean kg/m^2^ (95% CI)	32.7 (32.1; 33.3)
Abscess count, mean (95% CI)	2.9 (2.6; 3.3)
Inflammatory nodule count, mean (95% CI)	10.9 (10.1; 11.7)
Draining tunnel count, mean (95% CI)	3.2 (2.9; 3.5)
Proportion of female patients, mean % (95% CI)	58.6 (54.8; 62.4)
Proportion of current smokers, mean % (95% CI)	50.3 (46.7; 54.0)
Proportion of former smokers, mean % (95% CI)	14.6 (12.8; 16.5)
Hurley stage II, mean % (95% CI)^a^	56.1 (53.2; 59.0)
Hurley stage III, mean % (95% CI)^a^	42.5 (39.0; 46.0)
White race, mean % (95% CI)	79.2 (77.0; 81.3)
Black race, mean % (95% CI)	10.6 (8.0; 13.1)
Asian race, mean % (95% CI)	7.6 (5.0; 10.1)

Abbreviations: BMI, body mass index; CI, confidence interval.

^a^ SUNSHINE and SUNRISE also included patients with Hurley stage I.

### Hidradenitis Suppurativa Clinical Response 50

#### Primary analysis

**FIGURE 3:**
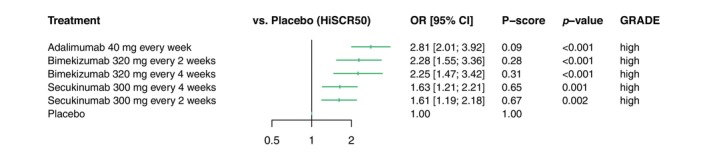
Forest plot for the primary network analysis on the Hidradenitis Suppurativa Clinical Response 50. Clinical response defined by HiSCR50 after 12–16 weeks of treatment is shown for all treatments in relation to placebo. The treatments are ranked according to *P* scores. The certainty of evidence is provided on the right (GRADE). CI, confidence interval; GRADE, GRADE Working Group approach for rating the quality of effect estimates from network meta‐analysis; HiSCR50, Hidradenitis Suppurativa Clinical Response 50; OR, odds ratio.

All three biologic agents were significantly more effective than placebo. Adalimumab injected every week in a dosage of 40 mg was ranked most effective with an OR of 2.81 (95% CI: 2.01, 3.92; *p* < 0.001) for reaching a HiSCR50, with placebo as a comparator. Bimekizumab injected every 2 weeks in a dosage of 320 mg was ranked second most effective with an OR of 2.28 (95% CI: 1.54, 3.36; *p* < 0.001) compared to placebo. Secukinumab injected every 4 weeks in a dosage of 300 mg was ranked fourth with an OR of 1.63 (95% CI: 1.21, 2.21; *p* = 0.001) compared to placebo (Figure 3).

Adalimumab injected every week in a dosage of 40 mg was significantly more effective than secukinumab 300 mg injected every 2 weeks (OR: 1.74; 95% CI: 1.11, 2.73; *p* = 0.016) and 4 weeks (OR: 1.72; 95% CI: 1.09, 2.70; *p* = 0.019). Other comparisons between biologic agents were non‐significant (Table 2).

**TABLE 2:** League table for the primary network analysis on the hidradenitis suppurativa clinical response 50.

	Vs. adalimumab 40 mg every week	Vs. bimekizumab 320 mg every 2 weeks	Vs. bimekizumab 320 mg every 4 weeks	vs. Placebo	vs. Secukinumab 300 mg every 2 weeks	Vs. secukinumab 300 mg every 4 weeks
Adalimumab 40 mg every week	NA	1.23 (95% CI: 0.74, 2.06; *p* = 0.425)	1.25 (95% CI: 0.73, 2.14; *p* = 0.415)	2.81 (95% CI: 2.01, 3.92; *p* = 0)	1.74 (95% CI: 1.11, 2.73; *p* = 0.016)	1.72 (95% CI: 1.09, 2.7; *p* = 0.019)
Bimekizumab 320 mg every 2 weeks	0.81 (95% CI: 0.49, 1.35; *p* = 0.425)	NA	1.02 (95% CI: 0.76, 1.35; *p* = 0.918)	2.28 (95% CI: 1.55, 3.36; *p* = 0)	1.41 (95% CI: 0.86, 2.31; *p* = 0.17)	1.39 (95% CI: 0.85, 2.28; *p* = 0.185)
Bimekizumab 320 mg every 4 weeks	0.8 (95% CI: 0.47, 1.37; *p* = 0.415)	0.99 (95% CI: 0.74, 1.31; *p* = 0.918)	NA	2.25 (95% CI: 1.47, 3.42; *p* = 0)	1.39 (95% CI: 0.83, 2.34; *p* = 0.213)	1.37 (95% CI: 0.82, 2.31; *p* = 0.23)
Placebo	0.36 (95% CI: 0.25, 0.5; *p* = 0)	0.44 (95% CI: 0.3, 0.65; *p* = 0)	0.45 (95% CI: 0.29, 0.68; *p* = 0)	NA	0.62 (95% CI: 0.46, 0.84; *p* = 0.002)	0.61 (95% CI: 0.45, 0.83; *p* = 0.001)
Secukinumab 300 mg every 2 weeks	0.57 (95% CI: 0.37, 0.9; *p* = 0.016)	0.71 (95% CI: 0.43, 1.16; *p* = 0.17)	0.72 (95% CI: 0.43, 1.21; *p* = 0.213)	1.61 (95% CI: 1.19, 2.18; *p* = 0.002)	NA	0.99 (95% CI: 0.74, 1.33; *p* = 0.936)
Secukinumab 300 mg every 4 weeks	0.58 (95% CI: 0.37, 0.91; *p* = 0.019)	0.72 (95% CI: 0.44, 1.17; *p* = 0.185)	0.73 (95% CI: 0.43, 1.22; *p* = 0.23)	1.63 (95% CI: 1.21, 2.21; *p* = 0.001)	1.01 (95% CI: 0.75, 1.36; *p* = 0.936)	NA

*Note*: Odds ratios for all treatment comparisons are shown.

Abbreviations: CI, confidence interval; NA, not applicable.

Grading of the certainty of evidence according to the GRADE approach is included in Figure 3. No effect estimate was downgraded in the certainty of evidence. Further details on the quality of evidence can be found in Appendix B.

#### Sensitivity analysis

In the sensitivity analysis, patients included in BE HEARD I and II and treated with a systemic antibiotic were only counted as non‐responders if the systemic antibiotic was used as rescue therapy for HS. Since fewer patients from BE HEARD I and II were counted as non‐responders in the sensitivity analysis, the effect of bimekizumab was expected to be higher, and this could be seen. Adalimumab injected every week in a dosage of 40 mg was still ranked as the most effective and significantly more effective than secukinumab 300 mg injected every 2 weeks (OR: 1.74; 95% CI: 1.11, 2.73; *p* = 0.016) and 4 weeks (OR: 1.72; 95% CI: 1.09, 2.70; *p* = 0.019). In contrast to the primary analysis, bimekizumab 320 mg injected every 2 weeks was significantly more effective than secukinumab 300 mg injected every 2 weeks (OR: 1.65; 95% CI: 1.01, 2.68; *p* = 0.045). Other comparisons between biologics were non‐significant (Figure B2 and Table B2).

### Adverse events

#### Primary analysis

**FIGURE 4:**
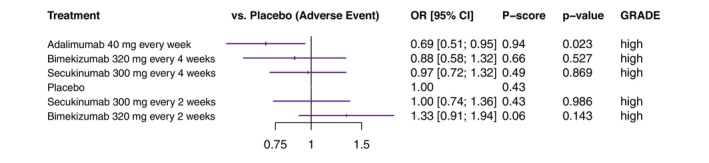
Forest plot for the primary network analysis on the occurrence of adverse events. The odds ratio for experiencing an adverse event is shown for all treatments with placebo as a comparator. The treatments are ranked according to *P* scores. The certainty of evidence is provided on the right (GRADE). CI, confidence interval; GRADE, GRADE Working Group approach for rating the quality of effect estimates from network meta‐analysis; OR, odds ratio.

Adalimumab injected every week in a dosage of 40 mg was ranked safest, with an OR for experiencing an AE of 0.69 (95% CI: 0.51, 0.95; *p* = 0.023) compared to placebo. Adalimumab was the only medication that showed significantly fewer AEs than placebo. Bimekizumab injected every 4 weeks in a dosage of 320 mg was ranked second safest, with an OR for experiencing an AE of 0.88 (95% CI: 0.58, 1.32; *p* = 0.527) compared to placebo. Secukinumab injected every 4 weeks in a dosage of 300 mg was ranked third safest, with an OR for experiencing an AE of 0.97 (95% CI: 0.72, 1.32; *p* = 0.869) compared to placebo (Figure 4).

Adalimumab injected every week in a dosage of 40 mg showed significantly fewer AEs than bimekizumab 320 mg injected every 2 weeks (odds ratio: 0.52; 95% CI: 0.32, 0.86; *p* = 0.010). Bimekizumab 320 mg injected every 2 weeks showed significantly more AEs than bimekizumab 320 mg injected every 4 weeks (odds ratio: 1.52; 95% CI: 1.13, 2.04; *p* = 0.006). Other comparisons between biologic agents were non‐significant (Table 3).

**TABLE 3:** League table for the primary network analysis on the occurrence of adverse events.

	Vs. adalimumab 40 mg every week	Vs. bimekizumab 320 mg every 2 weeks	Vs. bimekizumab 320 mg every 4 weeks	Vs. placebo	Vs. secukinumab 300 mg every 2 weeks	Vs. secukinumab 300 mg every 4 weeks
Adalimumab 40 mg every week	NA	0.52 (95% CI: 0.32, 0.86; *p* = 0.01)	0.79 (95% CI: 0.47, 1.33; *p* = 0.38)	0.69 (95% CI: 0.51, 0.95; *p* = 0.023)	0.69 (95% CI: 0.45, 1.07; *p* = 0.101)	0.71 (95% CI: 0.46, 1.1; *p* = 0.129)
Bimekizumab 320 mg every 2 weeks	1.91 (95% CI: 1.17, 3.13; *p* = 0.01)	NA	1.52 (95% CI: 1.13, 2.04; *p* = 0.006)	1.33 (95% CI: 0.91, 1.94; *p* = 0.143)	1.32 (95% CI: 0.81, 2.16; *p* = 0.259)	1.36 (95% CI: 0.84, 2.22; *p* = 0.213)
Bimekizumab 320 mg every 4 weeks	1.26 (95% CI: 0.75, 2.12; *p* = 0.38)	0.66 (95% CI: 0.49, 0.89; *p* = 0.006)	NA	0.88 (95% CI: 0.58, 1.32; *p* = 0.527)	0.87 (95% CI: 0.52, 1.46; *p* = 0.604)	0.9 (95% CI: 0.54, 1.5; *p* = 0.682)
Placebo	1.44 (95% CI: 1.05, 1.97; *p* = 0.023)	0.75 (95% CI: 0.52, 1.1; *p* = 0.143)	1.14 (95% CI: 0.76, 1.72; *p* = 0.527)	NA	1 (95% CI: 0.73, 1.35; *p* = 0.986)	1.03 (95% CI: 0.76, 1.39; *p* = 0.869)
Secukinumab 300 mg every 2 weeks	1.44 (95% CI: 0.93, 2.24; *p* = 0.101)	0.76 (95% CI: 0.46, 1.23; *p* = 0.259)	1.15 (95% CI: 0.69, 1.91; *p* = 0.604)	1 (95% CI: 0.74, 1.36; *p* = 0.986)	NA	1.03 (95% CI: 0.76, 1.4; *p* = 0.856)
Secukinumab 300 mg every 4 weeks	1.4 (95% CI: 0.91, 2.18; *p* = 0.129)	0.73 (95% CI: 0.45, 1.19; *p* = 0.213)	1.11 (95% CI: 0.67, 1.86; *p* = 0.682)	0.97 (95% CI: 0.72, 1.32; *p* = 0.869)	0.97 (95% CI: 0.72, 1.32; *p* = 0.856)	NA

*Note*: Odds ratios for all treatment comparisons are shown. The 95% CI is provided in brackets.

Abbreviations: CI, confidence interval; NA, not applicable.

Grading of the certainty of evidence according to the GRADE approach is shown in Figure 4. There was no downgrade in the certainty of evidence of an effect estimate. Further details on the quality of evidence can be found in Appendix C.

#### Sensitivity analysis

In the sensitivity analysis, worsening of HS was not counted as an AE, which may enable a more realistic and precise analysis of the occurrence of true drug‐associated AEs, as disease‐related events reported as AEs are at least partially accounted for. In contrast to the primary analysis, where adalimumab showed significantly fewer AEs than placebo, there was no significant difference of any biologic agent to placebo (Figure C2 and Table C2).

### Severe adverse events

No medication showed a significant difference compared to placebo in terms of SAE. Adalimumab injected every week in a dosage of 40 mg was ranked safest, with an OR for experiencing an SAE of 0.54 (95% CI: 0.20, 1.47; *p* = 0.225) compared to placebo. Secukinumab injected every 2 weeks in a dosage of 300 mg was ranked second safest, with an OR for experiencing a SAE of 0.80 (95% CI: 0.32, 1.99; *p* = 0.637) compared to placebo. Secukinumab injected every 4 weeks in the same dosage was ranked fourth safest, with an OR for experiencing a SAE of 4.25 (95% CI: 0.53, 34.31; *p* = 0.175) compared to placebo. All treatment comparisons were non‐significant (Figure D1 and Table D1).

Grading of the certainty of evidence according to the GRADE approach is shown in Figure D1. The effect estimates for adalimumab and secukinumab were downgraded to moderate due to considerable imprecision or wide confidence intervals because of few events in relation to the number of cases. The effect estimates for bimekizumab were downgraded to low due to high imprecision, also because of few events relative to the number of cases. More details on the quality of evidence can be found in Appendix D.

## DISCUSSION

The treatment of HS remains a challenge in clinical practice. Adalimumab was the first approved biologic agent for moderate‐to‐severe HS, and its approval has since been considered a breakthrough in HS treatment. However, a considerable percentage of patients are unable to achieve optimal treatment goals despite adalimumab treatment.^21^ Secukinumab, targeting IL‐17A, and bimekizumab, which targets both IL‐17A and IL‐17F, have met primary efficacy endpoints in phase III trials, have been approved, and enrich the therapeutic armamentarium available for moderate‐toto‐severe HS.^9^, ^10^

In our NMA comparing phase III RCTs of biologics in HS, adalimumab, secukinumab and bimekizumab showed a significantly better HiSCR50 response than placebo after 12–16 weeks. Adalimumab 40 mg weekly ranked as the most effective in reaching a HiSCR50 response, followed by bimekizumab 320 mg every 2 weeks, bimekizumab 320 mg every 4 weeks, secukinumab 300 mg every 4 weeks and secukinumab 300 mg every 2 weeks. Adalimumab was found to be significantly more effective than both doses of secukinumab, while no significant difference was found between adalimumab and both doses of bimekizumab or between bimekizumab and secukinumab. Regarding AEs and SAEs, none of the therapies bore significantly higher risks than placebo. The lowest rate of AEs was reported in the adalimumab 40 mg group, followed by bimekizumab 320 mg every 4 weeks, secukinumab 300 mg every 4 weeks, secukinumab 300 mg every 2 weeks and bimekizumab 320 mg every 2 weeks. Adalimumab 40 mg every week showed significantly less AEs than bimekizumab 320 mg every 2 weeks. Bimekizumab 320 mg every 4 weeks showed significantly less AEs than bimekizumab 320 mg every 2 weeks. Other comparisons between biologics were nonsignificant. Interestingly, the rate of AEs in patients treated with adalimumab 40 mg every week was significantly lower than in the placebo group. This is most likely due to the fact that in the clinical trials used for our NMA, worsening of HS was reported as an adverse effect and that worsening of HS may have been less common in the treatment group than in the placebo group. In fact, there was no significant difference between any biologic and placebo in a sensitivity analysis in which worsening of HS was not considered an AE. Adalimumab 40 mg every week also showed the fewest AEs in the sensitivity analysis. Finally, the rates of SAEs were slightly higher with both bimekizumab doses compared to placebo, although not significantly. All treatment comparisons for SAEs were nonsignificant.

Appendix E briefly summarizes previous NMAs that focused on systemic treatments for HS.^12^, ^13^, ^14^

None of these NMAs included data from the recently concluded phase III trial of bimekizumab, and all of these NMAs included trials at different phases, which resulted in a certain degree of heterogeneity in methodology and baseline characteristics among included studies. Our NMA included only phase III clinical trials to maximize the homogeneity of the methodology and thus the comparability of the included studies and interventions.

Our study does have some limitations. First, it is limited to the endpoint of 12–16 weeks. Long‐term efficacy and safety data still need further evaluation, especially given that HiSCR50 can be reached after 24 weeks if an earlier response was not achieved or only partially achieved as for the patients with dermal tunnels.^22^

Second, the analysed trials differed in terms of inclusion criteria. Mildly affected Hurley I patients were only included in SUNSHINE and SUNRISE. This could have reduced the difference between the treated and placebo groups in SUNSHINE and SUNRISE and thus could have reduced the effect estimates for the efficacy of secukinumab. This effect has been already described in clinical trials on psoriasis or atopic dermatitis.^23^, ^24^

Third, different methods for handling missing data have been applied by the included trials for the HiSCR50 efficacy endpoint. In PIONEER I and II, non‐response imputation was used. In contrast, multiple imputation was used in SUNSHINE, SUNRISE, BE HEARD I and BE HEARD II. Multiple imputations may have positively affected the effect estimates for the efficacy of bimekizumab and secukinumab compared to adalimumab.

Fourth, the included trials differed in terms of allowed background medication and in evaluating treatment efficacy when rescue antibiotic medication was administered. While in PIONEER I, patients receiving oral antibiotic agents for HS were required to stop treatment before baseline, patients included in PIONEER II, SUNSHINE, SUNRISE, BE HEARD I and BE HEARD II were allowed to continue treatment with antibiotics (tetracycline class) in stable dosages. Only in BE HEARD I and II, patients who took any systemic antibiotic, not only as rescue for HS, were treated as non‐responders. This may have favoured secukinumab and adalimumab in efficacy compared to bimekizumab. Therefore, we performed a sensitivity analysis in which for BE HEARD I and II, taking an antibiotic was considered a non‐response only if the antibiotic was taken for HS. In the sensitivity analysis, adalimumab was still ranked most effective and significantly more effective than secukinumab injected every 2 weeks and every 4 weeks. However, in contrast to the primary analysis, bimekizumab injected every 2 weeks was found to be significantly more effective than secukinumab injected every 2 weeks.

Finally, with regard to SAEs, the certainty of evidence was downgraded due to significant imprecision or wide confidence intervals. The low precision is due to the low number of SAEs that occurred during the limited observation periods of the included phase III studies. Post‐marketing phase IV surveillance studies with long observation periods are required to further assess the safety profile, in particular, the long‐term safety profile and the risk of SAEs.

## CONCLUSIONS AND FUTURE PERSPECTIVES

In this NMA that investigated the comparative efficacy and safety of biologic agents for the treatment of moderate‐to‐severe HS by including data of methodologically comparable phase III trials, adalimumab 40 mg every week was ranked most effective in achieving a HiSCR50 after 12–16 weeks, followed by bimekizumab 320 mg every 2 weeks, bimekizumab 320 mg every 4 weeks, secukinumab 300 mg every 4 weeks and secukinumab 300 mg every 2 weeks. Adalimumab 40 mg showed the fewest adverse events, followed by bimekizumab 320 mg every 4 weeks, secukinumab 300 mg every 4 weeks, secukinumab 300 mg every 2 weeks and bimekizumab 320 mg every 2 weeks.

These results combined with the extensive clinical experience support the role of adalimumab as ‘standard of care’ in the treatment of moderate‐to‐severe HS. However, it should be noted that these conclusions are based on a 16‐week observation period and that the potential long‐term superiority of adalimumab remains to be demonstrated.

Despite the undeniable progress in managing moderate‐to‐severe HS that biologic agents offer, a considerable proportion of HS patients with moderate‐to‐severe disease unfortunately do not reach a HiSCR50 within 12–16 weeks. It remains to be determined whether non‐responder patients would benefit from switching to another biologic, or if therapeutic agents targeting more than one cytokine/signaling pathway are required for optimal disease control in HS.

## AUTHOR CONTRIBUTIONS

Lars E. French, Laura Calabrese and Benjamin Kendziora conceived the presented idea. Benjamin Kendziora developed the theory and performed the computations. Alessandra Cartocci verified the analytical methods. Lars E. French and Pietro Rubegni encouraged Laura Calabrese and Benjamin Kendziora and supervised the findings of this work. All authors discussed the results and contributed to the final manuscript.

## FUNDING INFORMATION

This work was funded by the Munich Clinician Scientist Program (MCSP) of the Ludwig Maximilian University (LMU) (grant number CS 067) and by the European Union (EU)—Next Generation European Union, Mission 4 Component 2 Inv. 1.5 CUP B63C22000680007. The funders had no role in the study design; data collection, analysis and interpretation of data; writing of the report and decision to submit the article for publication. The researches were independent from funders.

## CONFLICT OF INTEREST STATEMENT

L.E.F. received financial support for his research group working on inflammatory dermatoses from AbbVie, Almirall, Amgen, Janssen, Klinge Pharma, Leo Pharma, Lilly, Novartis, UCB, Sanofi, Incyte and Celltrion; outside the submitted work. L.C. received honoraria for presentations from AbbVie and Almirall; outside the submitted work. No other relationships or activities that could appear to have influenced the submitted work.

## ETHICAL APPROVAL

Not applicable.

## Supporting information


Appendix S1

## Data Availability

The data sets generated and analysed during the current study are available from the corresponding author upon reasonable request. Access to the data will be granted for legitimate academic purposes.
