# Supplementary material for: Efficacy and safety of biologics for hidradenitis suppurativa: A network meta‐analysis of phase III trials
Source: J Eur Acad Dermatol Venereol. 2025 Mar 10;40(4):637–45. doi: 10.1111/jdv.20617 (PMC13014433; doi:10.1111/jdv.20617)
Supplement: Supplementary file 1 — Appendix S1 [file JDV-40-637-s001.pdf]

Appendix

Appendix A *2*
Screening of References 2
Data Extraction 2
Quality of Evidence 2
Performance of the Statistical Analysis and Software 2
Study Design and Use of Concomitant Antibiotics in the Included Studies 3
Table A1 4
Table A2 5
Table A3 13
Appendix B *14*
Quality of Evidence in the Primary Network Analysis on the Hidradenitis Suppurativa Clinical Response 50 14
Table B1 15
Table B2 16
Figure B1 17
Figure B2 18
Figure B3 19
Appendix C *20*
Quality of Evidence in the Primary Network Analysis on the Occurrence of Adverse Events 20
Table C1 21
Table C2 22
Figure C1 23
Figure C2 24
Figure C3 25
Appendix D *25*
Quality of Evidence in the Primary Network Analysis on the Occurence of Severe Adverse Events 25
Table D1 27
Table D2 28
Figure D1 29
Figure D2 30
Figure D3 31
Appendix E *32*
References *33*

Appendix A

Screening of References

Titles and abstracts of references revealed by the search strategy were screened independently by two reviewers (B.K. and L.C.). References identified as potentially eligible by at least one reviewer were checked for eligibility by reviewing the full text. Discrepancies between the two reviewers were solved by discussion.

Data Extraction

Two reviewers (B.K. and L.C.) independently extracted data from eligible studies onto a predefined extraction form, which was crosschecked for discrepancies. The treatment duration was defined as the time between first and last administration plus the time between doses. Engauge Digitizer, version 12.1, was used to extract data that was only presented as graphic illustration.

Quality of Evidence

Cochran's Q statistic was used to test for heterogeneity in the network (within designs). Net splitting was applied to evaluate inconsistency (between designs). The risk of bias in individual studies was evaluated for each outcome by two reviewers (B.K. and L.C.) using the revised Cochrane Risk of Bias Tool 2 for randomized trials. Discrepancies were solved by discussion (1). The risk of publication bias across studies was assessed for each outcome using Egger's regression test in a comparison-adjusted funnel plot. Finally, the GRADE Working Group approach was used to rate the certainty in all treatment effect estimates relative to placebo (2).

Performance of the Statistical Analysis and Software

The statistical calculations were carried out in R, version 4.2.2, in duplicate and independently by two reviewers (B.K. and A.C.), both of whom hold a Master's degree in statistics. The R packages meta, netmeta, robvis and ggplot2 R packages were used. An alpha level of 0.05 was set as the cut-off to achieve statistical significance. This corresponds to a 95% confidence interval (CI) of the odds ratio without the inclusion of 1.

Study Design and Use of Concomitant Antibiotics in the Included Studies

PIONEER I and II were similarly designed, multicentric phase III studies investigating the efficacy and safety of adalimumab in moderate-to-severe HS with two double-blind placebo-controlled periods. Period 1 was covered by the present NMA, where patients were randomly assigned in a 1:1 ratio to 40 mg of adalimumab weekly versus matching placebo for 12 weeks. While in PIONEER I, patients receiving oral antibiotic agents for HS were required to stop treatment for at least 28 days before baseline, patients included in PIONEER II were allowed to continue treatment with antibiotics (tetracycline class) in stable doses. The primary efficacy end point was clinical response at week 12, defined as HiSCR50.

SUNSHINE and SUNRISE were identical, multicentric, randomized, placebo-controlled, double-blind phase III trials evaluating secukinumab in moderate-to-severe HS. Treatment period 1 was covered by this NMA. In both trials, patients were randomly assigned (1:1:1) to receive subcutaneous secukinumab 300 mg every 2 weeks, subcutaneous secukinumab 300 mg every 4 weeks or subcutaneous placebo. Patients were allowed to continue treatment with antibiotics of the tetracycline class in stable doses. The primary efficacy endpoint was HiSCR50 at week 16.

BE HEARD I and BE HEARD II were identical, multicentric, randomized, placebo-controlled, double-blind phase III trials evaluating bimekizumab in moderate-to-severe HS. The initial treatment period was covered by this NMA. Patients were randomized (4:2:1) to receive subcutaneous bimekizumab 320 mg every 2 weeks, subcutaneous bimekizumab 320 mg every 4 weeks or subcutaneous placebo. Patients using a stable dose of a tetracycline class antibiotic for 28 days before baseline were allowed to continue antibiotics. HiSCR50 at week 16 was the primary efficacy endpoint.

Table A1

#	Searches	Results
1	“hidradenitis”.ab,kf,ti.	11374
2	“suppurativa”.ab,kf.ti.	10604
3	“random”.ab,kf.ti.	3370010
4	“phase”.ab,kf.ti.	2642298
5	“3”.ab,kf.ti.	11315586
6	“III”.ab,kf.ti.	1494300
7	“acne”.ab,kf.ti.	46644
8	“akne”.ab,kf.ti.	54
9	“inversa”.ab,kf.ti.	1885
10	“verneuil”.ab,kf.ti.	111
11	1 and 2	10440
12	5 or 6	12386760
13	4 and 12	855764
14	7 or 8	46672
15	9 and 14	1332
16	10 or 11 or 15	10732
17	3 and 13 and 16	69

Search Term. Search Term used in Ovid to search MEDLINE and Embase with results.

Table A2

Title	Source	Authors	Reason for exclusion
Secukinumab in moderate to severe hidradenitis suppurativa: Primary endpoint analysis from the SUNSHINE and SUNRISE Phase 3 trials.	Australasian Journal of Dermatology. Conference: 55th Australasian College of Dermatologists Annual Scientific Meeting. Sydney, NSW Australia. 64(Supplement 1) (pp 25-26), 2023. Date of Publication: May 2023.	Gottlieb A.B, et al.	Conference abstract
Race and Ethnicity Gaps in Global Hidradenitis Suppurativa Clinical Trials.	Dermatology (Basel, Switzerland). (pp 1-6), 2019. Date of Publication: 20 Dec 2019.	Price K.N, et al.	Exclusion criterion (not a phase III RCT)
Adalimumab Medium-Term Dosing Strategy in Moderate-to-Severe Hidradenitis Suppurativa: Integrated Results from the Phase 3, Randomized, Placebo-Controlled, PIONEER Trials.	The British journal of dermatology. (no pagination), 2019. Date of Publication: 27 Mar 2019.	Jemec G.B.E, et al.	Exclusion criterion (not a phase III RCT) - PostHoc analysis
Development and initial validation of the HS-IGA: a novel hidradenitis suppurativa-specific investigator global assessment for use in interventional trials*.	British Journal of Dermatology. 187(2) (pp 203-210), 2022. Date of Publication: August 2022.	Garg A, et al.	Exclusion criterion (not a phase III RCT)
Janus kinase 1 inhibitor INCB054707 for patients with moderate-to-severe hidradenitis suppurativa: results from two phase II studies*.	British Journal of Dermatology. 186(5) (pp 803-813), 2022. Date of Publication: May 2022.	Alavi A, et al.	Exclusion criterion (phase III RCT on a drug other than adalimumab, secukinumab and bimekizumab)
Successful treatment with high dosage infliximab after failure of IL-17 inhibitors: A series of 12 hidradenitis suppurativa's patients.	Experimental Dermatology. Conference: 11th European Hidradenitis Suppurativa e.V. Conference, EHSF 2022. Virtual. 31(Supplement 1) (pp 104-105), 2022. Date of Publication: September 2022.	Fougerousse A.C, et al.	Conference abstract
Outcome measures for moderate and severe hidradenitis suppurativa: Lessons learned from the SHINE study.	Experimental Dermatology. Conference: 11th European Hidradenitis Suppurativa e.V. Conference, EHSF 2022. Virtual. 31(Supplement 1) (pp 44), 2022. Date of Publication: September 2022.	Giamarellos-Bourboulis EJ, et al.	Conference abstract
Erratum: Efficacy and safety of bimekizumab in moderate to severe hidradenitis suppurativa: a phase 2, double-blind, placebo-controlled randomized clinical trial (JAMA Dermatol (2021) DOI: 10.1001/jamadermatol.2021.2905).	JAMA Dermatology. 157(11) (pp 1384), 2021. Date of Publication: November 2021.	Glatt S, et al.	Exclusion criterion (not a phase III RCT)
Efficacy and Safety of Adalimumab in Conjunction with Surgery in Moderate to Severe Hidradenitis Suppurativa: The SHARPS Randomized Clinical Trial.	JAMA Surgery. 156(11) (pp 1001-1009), 2021. Date of Publication: November 2021.	Bechara FG, et al.	Exclusion criterion (not a phase III RCT)
Race and Ethnicity Gaps in Global Hidradenitis Suppurativa Clinical Trials.	Dermatology. 237(1) (pp 97-102), 2021. Date of Publication: January 2021.	Price KN, et al.	Duplicate

LB782 A retrospective case series of Ustekinumab therapy in patients with severe and recalcitrant Hidradenitis Suppurativa.	Journal of Investigative Dermatology. Conference: SID 2021 Virtual Meeting Late-Breaking. Virtual, Online. 141(9) (pp B17), 2021. Date of Publication: September 2021.	Jiang SW, et al.	Conference abstract
LB791 Avacopan, a highly selective small molecule inhibitor of c5a receptor, in patients with Hidradenitis Suppurativa: Initial results from a randomized, double-blind, placebo-controlled, phase 2 study (aurora).	Journal of Investigative Dermatology. Conference: SID 2021 Virtual Meeting Late-Breaking. Virtual, Online. 141(9) (pp B19), 2021. Date of Publication: September 2021.	Kirby JS, et al.	Conference abstract
A randomized, placebo-controlled, phase 2 study of the Janus Kinase 1 inhibitor INCB054707 for patients with moderate-to-severe hidradenitis suppurativa.	Experimental Dermatology. Conference: 10th European Hidradenitis Suppurativa e.V. Conference, EHSF 2021. Virtual. 30(SUPPL 1) (pp 69-70), 2021. Date of Publication: June 2021.	Alavi A, et al.	Duplicate
American Academy of Dermatology 2020 AAD VMX.	Journal of the American Academy of Dermatology. Conference: American Academy of Dermatology 2020 AAD VMX. Virtual, Online. 83(6 Supplement) (pp A1-A4), 2020. Date of Publication: December 2020.	Anonymous	Conference abstract
Adalimumab in conjunction with surgery in patients with moderate to severe hidradenitis suppurativa: Baseline characteristics from a phase 4, double-blind, randomized, placebo-controlled study.	Experimental Dermatology. Conference: 9th Conference of the European Hidradenitis Suppurativa Foundation. Athens Greece. 29(SUPPL 1) (pp 38), 2020. Date of Publication: July 2020.	Bechara FG, et al.	Conference abstract
Efficacy and safety results from the SHARPS study: Phase 4, randomized, controlled trial of adalimumab plus surgery in moderate-to-severe hidradenitis suppurativa.	Experimental Dermatology. Conference: 9th Conference of the European Hidradenitis Suppurativa Foundation. Athens Greece. 29(SUPPL 1) (pp 37), 2020. Date of Publication: July 2020.	Bechara FG, et al.	Duplicate
Efficacy and safety of bimekizumab, a dual interleukin (IL)-17A and IL-17F inhibitor, for the treatment of moderate-to-severe hidradenitis suppurativa (HS): A 12-week, randomised, double-blind, placebo-controlled, Phase 2 study.	Experimental Dermatology. Conference: 9th Conference of the European Hidradenitis Suppurativa Foundation. Athens Greece. 29(SUPPL 1) (pp 31-32), 2020. Date of Publication: July 2020.	Jemec GBE, et al.	Conference abstract
Adalimumab medium-term dosing strategy in moderate-to-severe hidradenitis suppurativa: integrated results from the phase III randomized placebo-controlled PIONEER trials.	British Journal of Dermatology. 181(5) (pp 967-975), 2019. Date of Publication: 01 Nov 2019.	Jemec GBE, et al.	Duplicate
Design of a phase 2, multicenter, randomized, double-blind, placebo-controlled trial of 2 different dose regimens of iFX-1, a C5a Inhibitor, as an Add-On Therapy for Granulomatosis with Polyangiitis or Microscopic Polyangiitis.	Arthritis and Rheumatology. Conference: American College of Rheumatology/Association of Rheumatology Health Professionals Annual Scientific Meeting, ACR/ARHP 2019. Atlanta, GA United States. 71(Supplement 10) (pp 3002-3003), 2019. Date of Publication: October 2019.	Merkel P, et al.	Duplicate

IFX-1 in patients with moderate to severe hidradenitis suppurativa/acne inversa (HS): Baseline characteristics of a double-blind, randomized phase 2B dose-finding study (SHINE).	Experimental Dermatology. Conference: 8th European Hidradenitis Suppurativa Foundation Conference, EHSF 2019. Wroclaw Poland. 28(Supplement 2) (pp 25-26), 2019. Date of Publication: February 2019.	Giamarellos-Bourboulis EJ, et al.	Conference abstract
Patient-reported outcomes among patients with hidradenitis suppurativa experiencing different levels of clinical response: Integrated analysis from two clinical studies.	Journal of the American Academy of Dermatology. Conference: American Academy of Dermatology 2018 Annual Meeting. San Diego United States. 79(3 Supplement 1) (pp AB216), 2018. Date of Publication: 01 Sep 2018.	Tzellos T, et al.	Conference abstract
Effect of adalimumab treatment on metabolic parameters over 36 weeks: Integrated analysis from 2 phase 3 studies in patients with hidradenitis suppurativa.	Journal of the American Academy of Dermatology. Conference: American Academy of Dermatology 2018 Annual Meeting. San Diego United States. 79(3 Supplement 1) (pp AB116), 2018. Date of Publication: 01 Sep 2018.	Zouboulis C.C, et al.	Conference abstract
IDEAL conference 2018: No innovation without evaluation.	International Journal of Surgery. Conference: IDEAL conference 2018: No innovation without evaluation. Bristol United Kingdom. 59(Supplement 1) (no pagination), 2018. Date of Publication: November 2018.	Anonymous	Conference abstract
Adalimumab in conjunction with surgery in patients with moderate to severe Hidradenitis Suppurativa: Study design and baseline results from a phase 4, double-blind, randomized, placebo-controlled study.	Experimental Dermatology. Conference: 7th European Hidradenitis Suppurativa Foundation Congress, EHSF 2018. Rotterdam Netherlands. 27(Supplement 1) (pp 14), 2018. Date of Publication: May 2018.	Bechara F, et al.	Duplicate
Infection-free Clinical Response Among Patients With Hidradenitis Suppurativa Who Were Treated With Adalimumab: Results from Two Phase 3 Studies.	Wounds : a compendium of clinical research and practice. 29(11) (pp E98-E102), 2017. Date of Publication: 01 Nov 2017.	Giamarellos-Bourboulis EJ, et al.	Exclusion criterion (not a phase III RCT)
Safety and efficacy of anakinra in severe hidradenitis suppurativa a randomized clinical trial.	JAMA Dermatology. 152(1) (pp 52-59), 2016. Date of Publication: January 2016.	Tzanetakou V, et al.	Exclusion criterion (not a phase III RCT)
Use of the hurley staging system for the assessment of hidradenitis suppurativa disease severity in 2 phase 3 clinical trials.	Journal of Investigative Dermatology. Conference: 46th Annual Meeting of the European Society for Dermatological Research, ESDR 2016. Munich Germany. 136(9 Supplement 2) (pp S218), 2016. Date of Publication: September 2016.	Martorell A, et al.	Conference abstract
Therapeutic response guided dosing strategy to optimize long-term adalimumab treatment in patients with hidradenitis suppurativa: Integrated results from the PIONEER phase 3 trials.	Journal of Investigative Dermatology. Conference: 46th Annual Meeting of the European Society for Dermatological Research, ESDR 2016. Munich Germany. 136(9 Supplement 2) (pp	Gulliver W, et al.	Conference abstract

	S161), 2016. Date of Publication: September 2016.		
Safety of adalimumab dosed every week and every other week in patients with hidradenitis suppurativa or psoriasis.	Journal of the American Academy of Dermatology. Conference: 74th Annual Meeting of the American Academy of Dermatology. Washington, DC United States. Conference Publication: (var.pagings). 74(5 SUPPL. 1) (pp AB269), 2016. Date of Publication: May 2016.	Leonardi C, et al.	Conference abstract
Time to response in patients with moderate-to-severe hidradenitis suppurativa who were treated with adalimumab: Results from PIONEER I and PIONEER II.	Journal of the American Academy of Dermatology. Conference: 74th Annual Meeting of the American Academy of Dermatology. Washington, DC United States. Conference Publication: (var.pagings). 74(5 SUPPL. 1) (pp AB75), 2016. Date of Publication: May 2016.	Sobell J, et al.	Conference abstract
Risk of flare in patients with hidradenitis suppurativa treated with adalimumab for 12 weeks during PIONEER I and PIONEER II: Two phase 3, randomized, placebo-controlled trials.	Journal of the American Academy of Dermatology. Conference: 74th Annual Meeting of the American Academy of Dermatology. Washington, DC United States. Conference Publication: (var.pagings). 74(5 SUPPL. 1) (pp AB71), 2016. Date of Publication: May 2016.	Forman SB, et al.	Conference abstract
Progression of hidradenitis suppurativa: Outcomes of placebo-treated patients in a phase 3, randomized, placebo-controlled trial (PIONEER II).	Journal of the American Academy of Dermatology. Conference: 74th Annual Meeting of the American Academy of Dermatology. Washington, DC United States. Conference Publication: (var.pagings). 74(5 SUPPL. 1) (pp AB68), 2016. Date of Publication: May 2016.	Kimball AB, et al.	Conference abstract
Clinical meaningfulness of the hidradenitis suppurativa clinical response endpoint to assess inflammation and treatment response in 2 phase 3, randomized, placebo-controlled trials (PIONEER I & II).	Journal of the American Academy of Dermatology. Conference: 74th Annual Meeting of the American Academy of Dermatology. Washington, DC United States. Conference Publication: (var.pagings). 74(5 SUPPL. 1) (pp AB48), 2016. Date of Publication: May 2016.	Kimball A.B, et al.	Conference abstract
Adalimumab treatment is associated with a trend toward reduced need for acute surgical interventions in patients with moderate-to-severe hidradenitis suppurativa.	Journal of the American Academy of Dermatology. Conference: 74th Annual Meeting of the American Academy of Dermatology. Washington, DC United States. Conference Publication: (var.pagings). 74(5 SUPPL. 1) (pp AB41), 2016. Date of Publication: May 2016.	Zouboulis C, et al.	Conference abstract

Spotlight on adalimumab in the treatment of active moderate-to-severe hidradenitis suppurativa.	Clinical, Cosmetic and Investigational Dermatology. 9 (pp 367-372), 2016. Date of Publication: 19 Oct 2016.	Fotiadou C, et al.	Exclusion criterion (not a phase III RCT)
Safety and efficacy of adalimumab in patients with moderate to severe hidradenitis suppurativa: Results from first 12 weeks of PIONEER I, a Phase 3, randomized, placebo-controlled trial.	Journal of Clinical and Aesthetic Dermatology. Conference: Maui Derm 2015. Maui, HI United States. 8(5 Supplement 1) (pp S10-S11), 2015. Date of Publication: May 2015.	Kimball AB, et al.	Conference abstract
Efficacy and safety of Adalimumab in patients with moderate to severe hidradenitis suppurativa: Results from PIONEER II, a phase 3 randomised placebo-controlled trial.	Australasian Journal of Dermatology. Conference: 48th Annual Scientific Meeting of the Australasian College of Dermatologists. Adelaide, SA Australia. Conference Publication: (var.pagings). 56(SUPPL. 2) (pp 34-35), 2015. Date of Publication: May 2015.	Escudero Herra L, et al.	Conference abstract
Safety and efficacy of Adalimumab in patients with moderate to severe hidradenitis suppurativa: Results from first 12 weeks of PIONEER I, a phase 3, randomized, placebo-controlled trial.	Australasian Journal of Dermatology. Conference: 48th Annual Scientific Meeting of the Australasian College of Dermatologists. Adelaide, SA Australia. Conference Publication: (var.pagings). 56(SUPPL. 2) (pp 34), 2015. Date of Publication: May 2015.	Escudero Herra L, et al.	Duplicate
Adalimumab treatment is associated with a trend toward reduced need for acute surgical interventions in patients with moderate-to-severe hidradenitis suppurativa.	Journal of Investigative Dermatology. Conference: 45th Annual Meeting of the European Society for Dermatological Research. Rotterdam Netherlands. Conference Publication: (var.pagings). 135(SUPPL. 2) (pp S10), 2015. Date of Publication: September 2015.	Zouboulis CC, et al.	Duplicate
Safety and efficacy of adalimumab in patients with moderate to severe hidradenitis suppurativa: Results from first 12 weeks of PIONEER I, a phase 3, randomized, placebo-controlled trial.	Journal of the American Academy of Dermatology. Conference: 73rd Annual Meeting of the American Academy of Dermatology. San Francisco, CA United States. Conference Publication: (var.pagings). 72(5 SUPPL. 1) (pp AB60), 2015. Date of Publication: May 2015.	Kimball A, et al.	Duplicate
Efficacy and safety of adalimumab in patients with moderate to severe hidradenitis suppurativa: Results from PIONEER II, a phase 3, randomized, placebo-controlled trial.	Journal of the American Academy of Dermatology. Conference: 73rd Annual Meeting of the American Academy of Dermatology. San Francisco, CA United States. Conference Publication: (var.pagings). 72(5 SUPPL. 1) (pp AB45), 2015. Date of Publication: May 2015.	Jemec G, et al.	Duplicate
Adalimumab improves treatment satisfaction with medication (TS-M) in patients with moderate to severe hidradenitis suppurativa (HS) in a 12-week randomized controlled trial (PIONEER II).	Journal of the American Academy of Dermatology. Conference: 73rd Annual Meeting of the American Academy of Dermatology. San Francisco, CA United States. Conference Publication:	Jemec GBE, et al.	Conference abstract

	(var.pagings). 72(5 SUPPL. 1) (pp AB39), 2015. Date of Publication: May 2015.		
Safety and efficacy of adalimumab in patients with moderate to severe hidradenitis suppurativa: Results from first 12 Weeks of PIONEER I, a Phase 3, Randomized, placebo-controlled trial.	Journal of Investigative Dermatology. Conference: 44th Annual Meeting of the European Society for Dermatological Research. Copenhagen Denmark. Conference Publication: (var.pagings). 134(SUPPL. 2) (pp S36), 2014. Date of Publication: September 2014.	Kimball A, et al.	Duplicate
HUMIRA Improves Health-Related Quality of Life (HRQoL) in patients with moderate to severe Hidradenitis Suppurativa (HS): Results from the first 12 weeks of PIONEER I.	Journal of Investigative Dermatology. Conference: 44th Annual Meeting of the European Society for Dermatological Research. Copenhagen Denmark. Conference Publication: (var.pagings). 134(SUPPL. 2) (pp S34), 2014. Date of Publication: September 2014.	Armstrong A, et al.	Conference abstract
Adalimumab improves treatment satisfaction with medication (TS-M) in patients with moderate to severe Hidradenitis Suppurativa (HS) in a 12-week randomised controlled trial (PIONEER I).	Journal of Investigative Dermatology. Conference: 44th Annual Meeting of the European Society for Dermatological Research. Copenhagen Denmark. Conference Publication: (var.pagings). 134(SUPPL. 2) (pp S31), 2014. Date of Publication: September 2014.	Jemec GB, et al.	Duplicate
Efficacy results using a novel hidradenitis suppurativa endpoint, HiSCR (hidradenitis suppurativa clinical response), from the placebo-controlled phase of a phase 2 adalimumab study.	Journal of the American Academy of Dermatology. Conference: 72nd Annual Meeting of the American Academy of Dermatology. Denver, CO United States. Conference Publication: (var.pagings). 70(5 SUPPL. 1) (pp AB42), 2014. Date of Publication: May 2014.	Jemec G, et al.	Conference abstract
Adalimumab treatment is associated with pain reduction in patients with hidradenitis suppurativa, regardless of the presence of depression: Results from a phase II, randomized, placebo-controlled trial.	Journal of the American Academy of Dermatology. Conference: 72nd Annual Meeting of the American Academy of Dermatology. Denver, CO United States. Conference Publication: (var.pagings). 70(5 SUPPL. 1) (pp AB35), 2014. Date of Publication: May 2014.	Scheinfeld N, et al.	Conference abstract
Adalimumab treatment in women with moderate to severe hidradenitis suppurativa using a novel endpoint, HISCR (hidradenitis suppurativa clinical response): Analysis from the placebo-controlled portion of a phase ii, randomized, double-blind study.	Reproductive Sciences. Conference: 61st Annual Scientific Meeting of the Society for Gynecologic Investigation, SGI 2014. Florence Italy. Conference Publication: (var.pagings). 21(3 SUPPL. 1) (pp 220A-221A), 2014. Date of Publication: March 2014.	Gottlieb A, et al.	Conference abstract
Efficacy results using a novel hidradenitis suppurativa endpoint, hiscr (hidradenitis suppurativa clinical response), from the placebo-controlled phase of a phase 2 adalimumab study.	Annals of Emergency Medicine. Conference: American College of Emergency Physicians, ACEP Research Forum 2013. Seattle, WA United States. Conference Publication: (var.pagings).	Scheinfeld NS, et al.	Conference abstract

	62(4 SUPPL. 1) (pp S151), 2013. Date of Publication: October 2013.		
Validation of the hidradenitis suppurativa clinical response as a clinical endpoint for hidradenitis suppurativa treatment evaluation.	Journal of the American Academy of Dermatology. Conference: 71st Annual Meeting of the American Academy of Dermatology. Miami Beach, FL United States. Conference Publication: (var.pagings). 68(4 SUPPL. 1) (pp AB68), 2013. Date of Publication: April 2013.	Okun M, et al.	Conference abstract
Efficacy and safety of adalimumab treatment in women with moderate to severe hidradenitis suppurativa: Analysis from the placebo-controlled portion of a phase II, randomized, double-blind study.	Journal of the American Academy of Dermatology. Conference: 71st Annual Meeting of the American Academy of Dermatology. Miami Beach, FL United States. Conference Publication: (var.pagings). 68(4 SUPPL. 1) (pp AB49), 2013. Date of Publication: April 2013.	Gottlieb AB, et al.	Conference abstract
A novel hidradenitis suppurativa efficacy variable, HiSCR (hidradenitis suppurativa clinical response), is responsive to change with adalimumab therapy: Results of a phase II study.	Journal of the American Academy of Dermatology. Conference: 71st Annual Meeting of the American Academy of Dermatology. Miami Beach, FL United States. Conference Publication: (var.pagings). 68(4 SUPPL. 1) (pp AB40), 2013. Date of Publication: April 2013.	Kimball AB, et al.	Conference abstract
Efficacy and safety of adalimumab treatment in women with moderate to severe hidradenitis suppurativa: Analysis from the placebo-controlled portion of a phase ii, randomized, double-blind study.	International Journal of Gynecology and Obstetrics. Conference: 20th FIGO World Congress of Gynecology and Obstetrics. Rome Italy. Conference Publication: (var.pagings). 119(SUPPL. 3) (pp S360), 2012. Date of Publication: October 2012.	Gottlieb AB, et al.	Conference abstract
Impact of weight and body mass index on high-sensitivity C-reactive protein response to adalimumab in hidradenitis suppurativa patients.	Journal of the American Academy of Dermatology. Conference: 70th Annual Meeting of the American Academy of Dermatology. San Diego, CA United States. Conference Publication: (var.pagings). 66(4 SUPPL. 1) (pp AB53), 2012. Date of Publication: April 2012.	Zouboulis C, et al.	Conference abstract
Adalimumab reduces pain in patients with hidradenitis suppurativa: Results from a placebo-controlled phase II trial.	Journal of the American Academy of Dermatology. Conference: 70th Annual Meeting of the American Academy of Dermatology. San Diego, CA United States. Conference Publication: (var.pagings). 66(4 SUPPL. 1) (pp AB42), 2012. Date of Publication: April 2012.	Kimball AB, et al.	Duplicate

Efficacy and safety of adalimumab in treatment of moderate to severe hidradenitis suppurativa: Results from the placebo-controlled portion of a phase II, randomized, double-blind study.	British Journal of Dermatology. Conference: 91st Annual Meeting of the British Association of Dermatologists. London United Kingdom. Conference Publication: (var.pagings). 165(SUPPL. 1) (pp 6), 2011. Date of Publication: July 2011.	Kimball AB, et al.	Conference abstract
A prospective clinical trial of open-label etanercept for the treatment of hidradenitis suppurativa.	Journal of the American Academy of Dermatology. 60(4) (pp 565-573), 2009. Date of Publication: April 2009.	Lee RA, et al.	Exclusion criterion (not a phase III RCT)
Guselkumab for the Treatment of Patients with Moderate-to-severe Hidradenitis Suppurativa: A Phase 2 Randomized Study.	Journal of the European Academy of Dermatology & Venereology. 2023 Jun 14	Kimball AB, et al.	Exclusion criterion (not a phase III RCT)
Development and initial validation of the HS-IGA: a novel hidradenitis suppurativa-specific investigator global assessment for use in interventional trials.	British Journal of Dermatology. 187(2):203-210, 2022 08.	Garg A, et al.	Duplicate
Janus kinase 1 inhibitor INCB054707 for patients with moderate-to-severe hidradenitis suppurativa: results from two phase II studies.	British Journal of Dermatology. 186(5):803-813, 2022 05.	Alavi A, et al.	Duplicate
Efficacy and Safety of Adalimumab in Conjunction With Surgery in Moderate to Severe Hidradenitis Suppurativa: The SHARPS Randomized Clinical Trial.	JAMA Surgery. 156(11):1001-1009, 2021 11 01.	Bechara FG, et al.	Duplicate
rf	Dermatology. 237(1):97-102, 2021.	Price K, et al.	Duplicate
Adalimumab medium-term dosing strategy in moderate-to-severe hidradenitis suppurativa: integrated results from the phase III randomized placebo-controlled PIONEER trials.	British Journal of Dermatology. 181(5):967-975, 2019 11.	Jemec GBE, et al.	Duplicate
Infection-free Clinical Response Among Patients With Hidradenitis Suppurativa Who Were Treated With Adalimumab: Results from Two Phase 3 Studies.	Wounds-A Compendium of Clinical Research & Practice. 29(11):E98-E102, 2017 Nov.	Giamarellos-Bourboulis EJ, et al.	Duplicate
Safety and Efficacy of Anakinra in Severe Hidradenitis Suppurativa: A Randomized Clinical Trial.	JAMA Dermatology. 152(1):52-59, 2016 Jan.	Tzanetakou V, et al.	Duplicate
Spotlight on adalimumab in the treatment of active moderate-to-severe hidradenitis suppurativa. [Review]	Clinical, Cosmetic and Investigational Dermatology CCID. 9:367-372, 2016.	Fotiadou C, et al.	Exclusion criterion (not a phase III RCT)
A prospective clinical trial of open-label etanercept for the treatment of hidradenitis suppurativa.	Journal of the American Academy of Dermatology. 60(4):565-73, 2009 Apr.	Lee RA, et al.	Duplicate

Excluded References. The table lists all excluded references with the reasons for exclusion.

Table A3

Study	Intervention	Randomized patients, n	Age, mean	Age, SD	Age, median	Age, IQR	BMI, mean	BMI, SD	Abscess count, mean	Abscesses, n	Inflammatory nodules, n	Inflammatory nodules, SD	Draining fistulae, n	Draining fistulae, SD	Females, n	Current smokers, n	Former smokers, n	Hurley 1, n	Hurley 2, n	Hurley 3, n	White race, n	Black race, n	Asian race, n	Other race, n
PIONEER I	Placebo	154	37.8	7.9	NA	NA	34.5	7.9	2.7	3.7	11.6	13.9	3.8	4.4	105	NA	NA	0	81	73	118	29		7
PIONEER I	Adalimumab 40 mg every week	153	36.2	7.6	NA	NA	33	7.6	2.8	3.5	11.5	10.9	4.6	5.2	91	NA	NA	0	80	73	116	33		4
PIONEER II	Placebo	163	36.1	12.2	NA	NA	32.9	7.9	2.4	3.3	9.4	9.6	3.7	5.2	113	NA	NA	0	89	74	130	20		13
PIONEER II	Adalimumab 40 mg every week	163	34.9	10	NA	NA	31.3	7.4	2	2.6	8.6	6.9	3	4.1	108	NA	NA	0	86	77	143	9		11
SUNSHINE	Secukinumab 300 mg every 2 weeks	181	37.1	12.5	NA	NA	32.6	7.9	2.9	4.3	10.1	7.8	2.9	3.4	102	95	26	7	104	70	145	15	19	2
SUNSHINE	Secukinumab 300 mg every 4 weeks	180	35.7	11.7	NA	NA	32.8	7.9	2.7	4	9.9	7.6	2.5	3.5	100	96	28	10	107	63	146	10	23	1
SUNSHINE	Placebo	180	35.5	10.8	NA	NA	32	7.1	2.7	3.8	10.1	7	2.4	3.2	102	101	30	8	121	51	139	12	24	5
SUNRISE	Secukinumab 300 mg every 2 weeks	180	37.3	11.5	NA	NA	31.9	7.8	3.9	5.4	10	7.7	3	3.6	98	97	32	6	92	82	133	18	16	13
SUNRISE	Secukinumab 300 mg every 4 weeks	180	35.5	11.4	NA	NA	32	7.5	2.9	4.1	10.4	7.6	2.5	3.5	103	90	25	6	106	68	139	19	16	6
SUNRISE	Placebo	183	36.2	11.3	NA	NA	31.4	7.4	3.2	5	9.6	6.8	2.6	3.2	105	106	24	3	110	70	143	12	19	9
BE HEARD I	Placebo	72	NA	NA	33.5	26-46	32.4	7.8	2.9	6.6	12.2	10	3.2	4	44	37	7	0	34	38	55	8	3	6
BE HEARD I	Bimekizumab 320 mg every 4 weeks	144	NA	NA	35	27-45	35.4	8.1	4.5	8.4	13.3	22.4	3.8	4.9	98	53	28	0	71	73	105	21	3	15
BE HEARD I	Bimekizumab 320 mg every 2 weeks	289	NA	NA	36	26-46	33.4	8.3	3.7	6.1	11.6	11.4	4	4.9	176	127	43	0	149	140	233	41	2	13
BE HEARD II	Placebo	74	NA	NA	37	28-47	33.8	8.7	2.4	2.8	11.4	6.7	3.5	3.7	31	38	10	0	45	29	64	5	5	0
BE HEARD II	Bimekizumab 320 mg every 4 weeks	144	NA	NA	33	26-42.5	32.2	7.5	3.5	5	14.1	13.3	2.8	3.1	77	73	14	0	89	55	119	13	7	5
BE HEARD II	Bimekizumab 320 mg every 2 weeks	291	NA	NA	35	27-45	32	8	3.3	5.9	13.4	12.2	3.6	4	150	134	49	0	177	114	232	22	22	15

Baseline Characteristics for Each Study. The table shows the baseline characteristics for the patient cohorts of each individual study. SD = standard deviation; IQR = interquartile range; BMI = body mass index.

Appendix B

Quality of Evidence in the Primary Network Analysis on the Hidradenitis Suppurativa Clinical Response 50

Cochran's Q statistic showed no significant heterogeneity (Q = 4.75; df = 5; p = 0.447). Net splitting did not show any inconsistency in the network (Appendix B Table B1). Appendix B Figure B1 displays the judgement on the risk of bias in the trials included in the NMA. No concerns were found. Egger's regression test in a comparison-adjusted funnel plot did not show publication bias across studies (p = 0.868). Figure B3 shows the comparison-adjusted funnel plot. Grading of the certainty of evidence according to the GRADE approach is included in Figure 3. No effect estimate was downgraded in the certainty of evidence.

Table B1

Comparison	Prop	Combined	Direct	Indirect	Diff	p-value
Adalimumab 40 mg every week vs Bimekizumab 320 mg every 2 weeks	0	0.21	NA	0.21	NA	NA
Adalimumab 40 mg every week vs Bimekizumab 320 mg every 4 weeks	0	0.22	NA	0.22	NA	NA
Adalimumab 40 mg every week vs Placebo	1	1.03	1.03	NA	NA	NA
Adalimumab 40 mg every week vs Secukinumab 300 mg every 2 weeks	0	0.55	NA	0.55	NA	NA
Adalimumab 40 mg every week vs Secukinumab 300 mg every 4 weeks	0	0.54	NA	0.54	NA	NA
Bimekizumab 320 mg every 2 weeks vs Bimekizumab 320 mg every 4 weeks	1	0.01	0.01	NA	NA	NA
Bimekizumab 320 mg every 2 weeks vs Placebo	1	0.82	0.82	NA	NA	NA
Bimekizumab 320 mg every 2 weeks vs Secukinumab 300 mg every 2 weeks	0	0.34	NA	0.34	NA	NA
Bimekizumab 320 mg every 2 weeks vs Secukinumab 300 mg every 4 weeks	0	0.33	NA	0.33	NA	NA
Bimekizumab 320 mg every 4 weeks vs Placebo	1	0.81	0.81	NA	NA	NA
Bimekizumab 320 mg every 4 weeks vs Secukinumab 300 mg every 2 weeks	0	0.33	NA	0.33	NA	NA
Bimekizumab 320 mg every 4 weeks vs Secukinumab 300 mg every 4 weeks	0	0.32	NA	0.32	NA	NA
Secukinumab 300 mg every 2 weeks vs Placebo	1	0.48	0.48	NA	NA	NA
Secukinumab 300 mg every 4 weeks vs Placebo	1	0.49	0.49	NA	NA	NA
Secukinumab 300 mg every 2 weeks vs Secukinumab 300 mg every 4 weeks	1	-0.01	-0.01	NA	NA	NA

Net Splitting for the Primary Network Analysis on the Hidradenitis Suppurativa Clinical Response 50. Net splitting did not show any inconsistency in the network since either direct or indirect evidence was used in the model, as can be seen in the second column. Prop = direct evidence proportion used for estimating the treatment effect in the network meta-analysis; Combined = estimated treatment effect in the network meta-analysis; Direct = estimated treatment effect derived from direct evidence; Indirect = estimated treatment effect derived from indirect evidence; Diff = difference between direct and indirect effect estimate; p-value = p-value of test for disagreement between direct and indirect effect estimate; NA = not applicable.

Table B2

	vs. Adalimumab 40 mg every week	vs. Bimekizumab 320 mg every 2 weeks	vs. Bimekizumab 320 mg every 4 weeks	vs. Placebo	vs. Secukinumab 300 mg every 2 weeks	vs. Secukinumab 300 mg every 4 weeks
Adalimumab 40 mg every week	NA	1.06 (95% CI: 0.64, 1.76; p=0.829)	1.1 (95% CI: 0.64, 1.87; p=0.738)	2.81 (95% CI: 2.01, 3.92; p=0)	1.74 (95% CI: 1.11, 2.73; p=0.016)	1.72 (95% CI: 1.09, 2.7; p=0.019)
Bimekizumab 320 mg every 2 weeks	0.95 (95% CI: 0.57, 1.57; p=0.829)	NA	1.04 (95% CI: 0.78, 1.38; p=0.808)	2.66 (95% CI: 1.81, 3.89; p=0)	1.65 (95% CI: 1.01, 2.68; p=0.045)	1.63 (95% CI: 1, 2.65; p=0.051)
Bimekizumab 320 mg every 4 weeks	0.91 (95% CI: 0.54, 1.56; p=0.738)	0.97 (95% CI: 0.73, 1.28; p=0.808)	NA	2.56 (95% CI: 1.69, 3.89; p=0)	1.59 (95% CI: 0.95, 2.66; p=0.078)	1.57 (95% CI: 0.94, 2.63; p=0.086)
Placebo	0.36 (95% CI: 0.25, 0.5; p=0)	0.38 (95% CI: 0.26, 0.55; p=0)	0.39 (95% CI: 0.26, 0.59; p=0)	NA	0.62 (95% CI: 0.46, 0.84; p=0.002)	0.61 (95% CI: 0.45, 0.83; p=0.001)
Secukinumab 300 mg every 2 weeks	0.57 (95% CI: 0.37, 0.9; p=0.016)	0.61 (95% CI: 0.37, 0.99; p=0.045)	0.63 (95% CI: 0.38, 1.05; p=0.078)	1.61 (95% CI: 1.19, 2.18; p=0.002)	NA	0.99 (95% CI: 0.74, 1.33; p=0.936)
Secukinumab 300 mg every 4 weeks	0.58 (95% CI: 0.37, 0.91; p=0.019)	0.62 (95% CI: 0.38, 1; p=0.051)	0.64 (95% CI: 0.38, 1.07; p=0.086)	1.63 (95% CI: 1.21, 2.21; p=0.001)	1.01 (95% CI: 0.75, 1.36; p=0.936)	NA

League Table for the Sensitivity Network Analysis on the Hidradenitis Suppurativa Clinical Response 50. Odds ratios for all treatment comparisons are shown. CI = confidence interval; NA = not applicable.

Figure B1

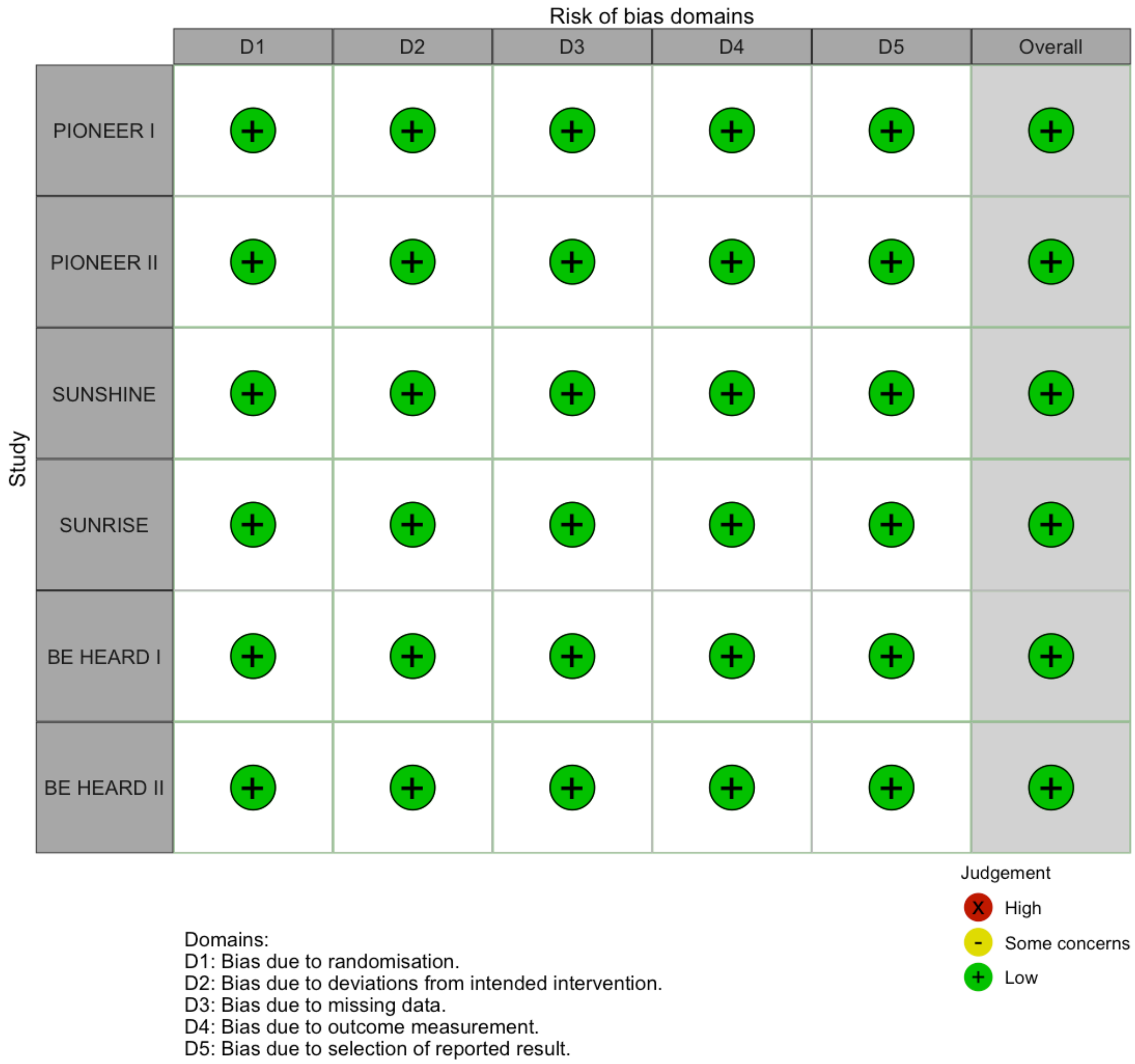

Risk of Bias Assessment for the Hidradenitis Suppurativa Clinical Response 50. The judgements for each trial providing data for the network meta-analysis on the HiSCR50 are shown in this traffic lights plot. HiSCR50 = Hidradenitis Suppurativa Clinical Response 50.

Figure B2

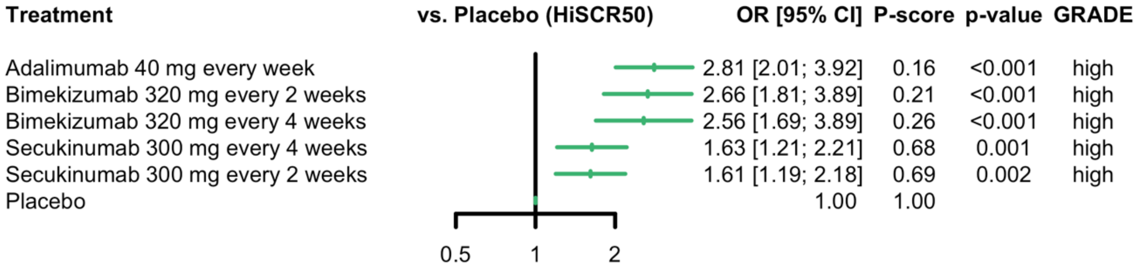

Forest Plot for the Sensitivity Network Analysis on the Hidradenitis Suppurativa Clinical Response 50. Clinical response defined by HiSCR50 after 12-16 weeks of treatment is shown for all treatments in relation to placebo. The treatments are ranked according to P-scores. The certainty of evidence is provided on the right. HiSCR50 = Hidradenitis Suppurativa Clinical Response 50; OR = odds ratio; CI = confidence interval; GRADE = GRADE Working Group approach for rating the quality of effect estimates from network meta-analysis.

Figure B3

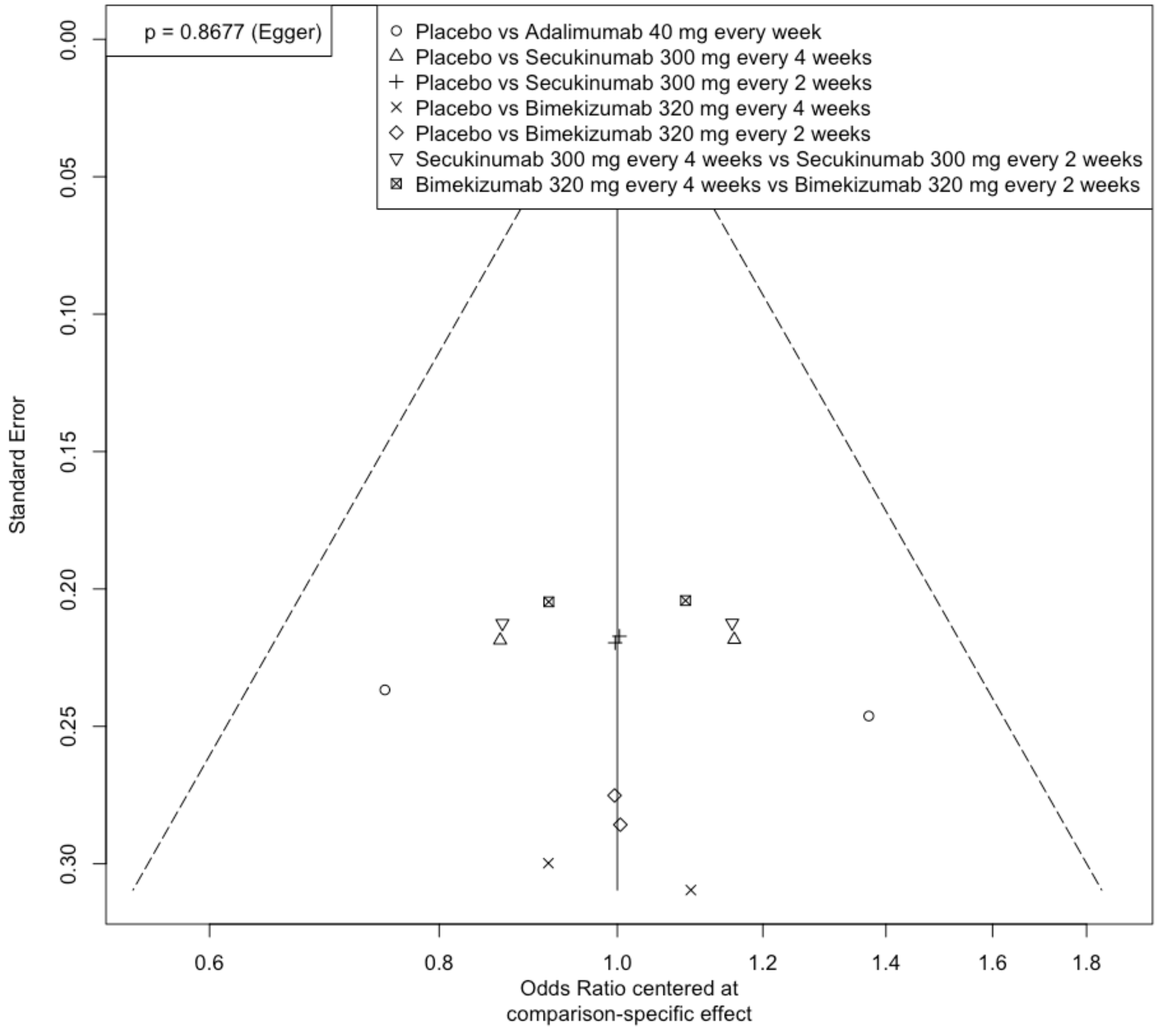

Comparison-Adjusted Funnel Plot for Network Analysis on the Hidradenitis Suppurativa Clinical Response 50. The p-value of Egger's test is shown in the top left-hand corner.

Appendix C

Quality of Evidence in the Primary Network Analysis on the Occurrence of Adverse Events

Cochran's Q statistic revealed no significant heterogeneity (Q = 0.7; df = 5; p = 0.983). Net splitting did not show any inconsistency in the network (Appendix C Table C1). The risk of bias assessment in individual studies is displayed in Appendix C Figure C1. There were no concerns. Egger's regression test in a comparison-adjusted funnel plot did not reveal publication bias across studies (p = 0.962). Figure C3 shows the comparison-adjusted funnel plot. Grading of the certainty of evidence according to the GRADE approach is shown in Figure 4. There was no downgrade in the certainty of evidence of an effect estimate.

Table C1

Comparison	Prop	Combined	Direct	Indirect	Diff	p-value
Adalimumab 40 mg every week vs Bimekizumab 320 mg every 2 weeks	0	-0.65	NA	-0.65	NA	NA
Adalimumab 40 mg every week vs Bimekizumab 320 mg every 4 weeks	0	-0.23	NA	-0.23	NA	NA
Adalimumab 40 mg every week vs Placebo	1	-0.37	-0.37	NA	NA	NA
Adalimumab 40 mg every week vs Secukinumab 300 mg every 2 weeks	0	-0.37	NA	-0.37	NA	NA
Adalimumab 40 mg every week vs Secukinumab 300 mg every 4 weeks	0	-0.34	NA	-0.34	NA	NA
Bimekizumab 320 mg every 2 weeks vs Bimekizumab 320 mg every 4 weeks	1	0.42	0.42	NA	NA	NA
Bimekizumab 320 mg every 2 weeks vs Placebo	1	0.28	0.28	NA	NA	NA
Bimekizumab 320 mg every 2 weeks vs Secukinumab 300 mg every 2 weeks	0	0.28	NA	0.28	NA	NA
Bimekizumab 320 mg every 2 weeks vs Secukinumab 300 mg every 4 weeks	0	0.31	NA	0.31	NA	NA
Bimekizumab 320 mg every 4 weeks vs Placebo	1	-0.13	-0.13	NA	NA	NA
Bimekizumab 320 mg every 4 weeks vs Secukinumab 300 mg every 2 weeks	0	-0.14	NA	-0.14	NA	NA
Bimekizumab 320 mg every 4 weeks vs Secukinumab 300 mg every 4 weeks	0	-0.11	NA	-0.11	NA	NA
Secukinumab 300 mg every 2 weeks vs Placebo	1	0	0	NA	NA	NA
Secukinumab 300 mg every 4 weeks vs Placebo	1	-0.03	-0.03	NA	NA	NA
Secukinumab 300 mg every 2 weeks vs Secukinumab 300 mg every 4 weeks	1	0.03	0.03	NA	NA	NA

Net Splitting for the Primary Network Analysis on the Occurrence of Adverse Events. Net splitting did not show any inconsistency in the network since either direct or indirect evidence was used in the model, as can be seen in the second column. Prop = direct evidence proportion used for estimating the treatment effect in the network meta-analysis; Combined = estimated treatment effect in the network meta-analysis; Direct = estimated treatment effect derived from direct evidence; Indirect = estimated treatment effect derived from indirect evidence; Diff = difference between direct and indirect effect estimate; p-value = p-value of test for disagreement between direct and indirect effect estimate; NA = not applicable.

Table C2

	vs. Adalimumab 40 mg every week	vs. Bimekizumab 320 mg every 2 weeks	vs. Bimekizumab 320 mg every 4 weeks	vs. Placebo	vs. Secukinumab 300 mg every 2 weeks	vs. Secukinumab 300 mg every 4 weeks
Adalimumab 40 mg every week	NA	0.59 (95% CI: 0.36, 0.96; p=0.034)	0.9 (95% CI: 0.54, 1.51; p=0.696)	0.85 (95% CI: 0.61, 1.17; p=0.309)	0.7 (95% CI: 0.45, 1.08; p=0.106)	0.68 (95% CI: 0.44, 1.06; p=0.09)
Bimekizumab 320 mg every 2 weeks	1.7 (95% CI: 1.04, 2.77; p=0.034)	NA	1.53 (95% CI: 1.15, 2.05; p=0.004)	1.43 (95% CI: 0.99, 2.07; p=0.054)	1.18 (95% CI: 0.74, 1.89; p=0.484)	1.16 (95% CI: 0.73, 1.86; p=0.533)
Bimekizumab 320 mg every 4 weeks	1.11 (95% CI: 0.66, 1.86; p=0.696)	0.65 (95% CI: 0.49, 0.87; p=0.004)	NA	0.94 (95% CI: 0.63, 1.4; p=0.749)	0.77 (95% CI: 0.47, 1.27; p=0.308)	0.76 (95% CI: 0.46, 1.25; p=0.276)
Placebo	1.18 (95% CI: 0.86, 1.64; p=0.309)	0.7 (95% CI: 0.48, 1.01; p=0.054)	1.07 (95% CI: 0.72, 1.59; p=0.749)	NA	0.82 (95% CI: 0.61, 1.11; p=0.199)	0.81 (95% CI: 0.6, 1.09; p=0.161)
Secukinumab 300 mg every 2 weeks	1.44 (95% CI: 0.93, 2.23; p=0.106)	0.85 (95% CI: 0.53, 1.35; p=0.484)	1.3 (95% CI: 0.79, 2.13; p=0.308)	1.21 (95% CI: 0.9, 1.63; p=0.199)	NA	0.98 (95% CI: 0.73, 1.32; p=0.904)
Secukinumab 300 mg every 4 weeks	1.46 (95% CI: 0.94, 2.27; p=0.09)	0.86 (95% CI: 0.54, 1.38; p=0.533)	1.32 (95% CI: 0.8, 2.17; p=0.276)	1.24 (95% CI: 0.92, 1.66; p=0.161)	1.02 (95% CI: 0.76, 1.37; p=0.904)	NA

League Table for the Sensitivity Network Analysis on the Occurrence of Adverse Events. Odds ratios for all treatment comparisons are shown. CI = confidence interval; NA = not applicable.

Figure C1

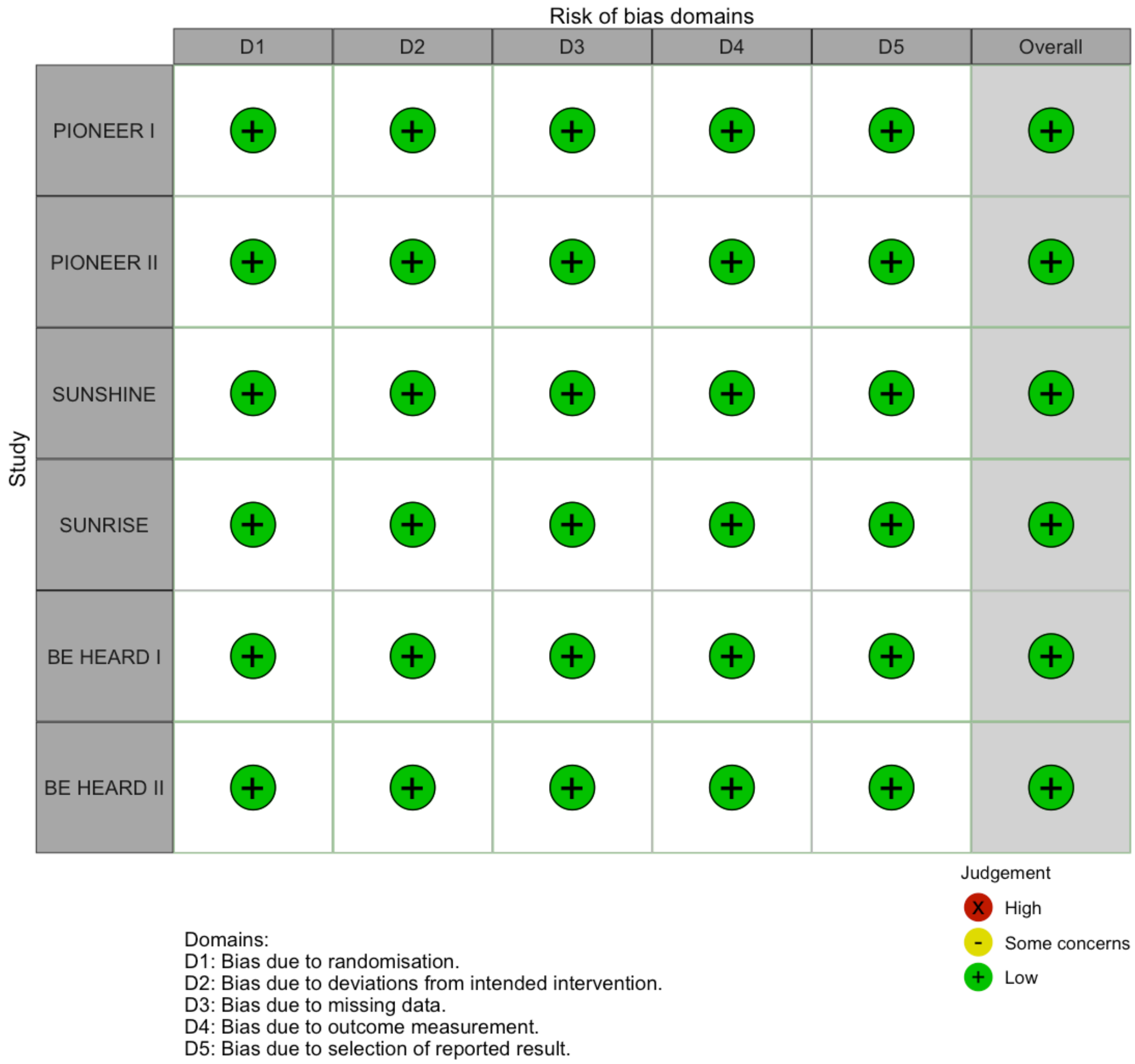

Risk of Bias Assessment for Adverse Events. The judgements for each trial providing data for the network meta-analysis on adverse events are shown in this traffic lights plot.

Figure C2

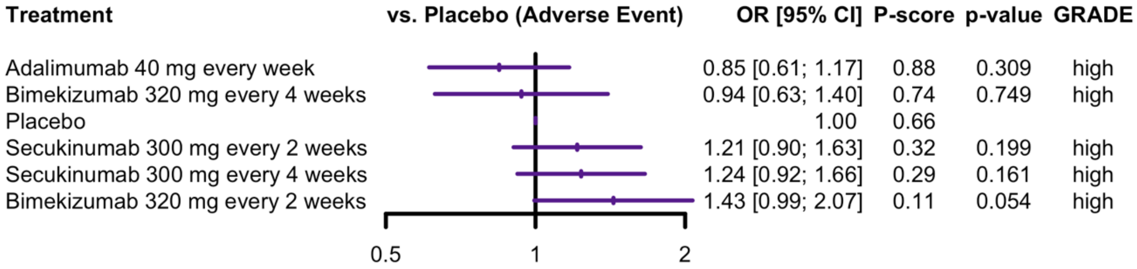

Forest Plot for the Sensitivity Network Analysis on the Occurrence of Adverse Events. The odds ratio for experiencing an adverse event other than worsening of HS is shown for all treatments with placebo as comparator. The treatments are ranked according to P-scores. The certainty of evidence is provided on the right. OR = odds ratio; CI = confidence interval; GRADE = GRADE Working Group approach for rating the quality of effect estimates from network meta-analysis; HS = hidradenitis suppurativa.

Figure C3

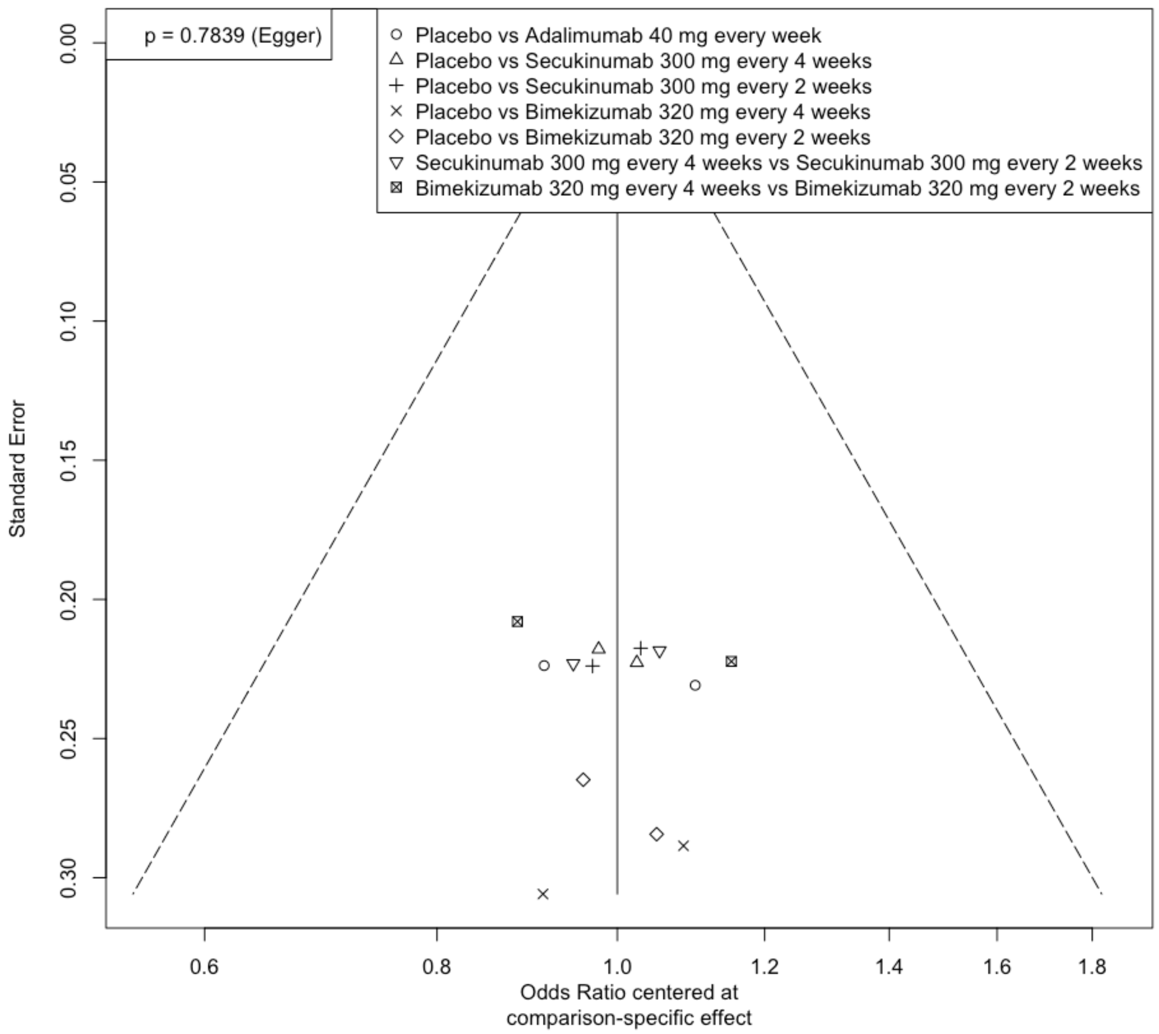

Comparison-Adjusted Funnel Plot for Network Analysis on the Occurrence of Adverse Events. The p-value of Egger's test is shown in the top left-hand corner.

Appendix D

Quality of Evidence in the Primary Network Analysis on the Occurence of Severe Adverse Events

Cochran's Q statistic revealed no significant heterogeneity (Q = 1.8; df = 5; p = 0.881). Net splitting did not show any inconsistency in the network (Appendix D Table D2). The risk of bias assessment in individual studies is displayed in Appendix D Figure D2. There were no concerns. Egger's regression test in a comparison-adjusted funnel plot did not show publication bias across studies (p = 0.962). Figure D3 shows the comparison-adjusted funnel plot. Grading

of the certainty of evidence according to the GRADE approach is shown in Appendix Figure D1. The effect estimates for adalimumab and secukinumab were downgraded to moderate due to considerable imprecision or wide confidence intervals as a result of few events in relation to the number of cases. The effect estimates for bimekizumab were downgraded to low due to high imprecision, also as a result of few events relative to the number of cases.

Table D1

	vs. Adalimumab 40 mg every week	vs. Bimekizumab 320 mg every 2 weeks	vs. Bimekizumab 320 mg every 4 weeks	vs. Placebo	vs. Secukinumab 300 mg every 2 weeks	vs. Secukinumab 300 mg every 4 weeks
Adalimumab 40 mg every week	NA	0.13 (95% CI: 0.01, 1.21; p=0.072)	0.13 (95% CI: 0.01, 1.28; p=0.08)	0.54 (95% CI: 0.2, 1.47; p=0.225)	0.67 (95% CI: 0.17, 2.59; p=0.558)	0.67 (95% CI: 0.17, 2.58; p=0.556)
Bimekizumab 320 mg every 2 weeks	8 (95% CI: 0.83, 77.14; p=0.072)	NA	1.01 (95% CI: 0.42, 2.41; p=0.985)	4.28 (95% CI: 0.56, 32.61; p=0.16)	5.33 (95% CI: 0.58, 49.26; p=0.14)	5.32 (95% CI: 0.58, 49.16; p=0.141)
Bimekizumab 320 mg every 4 weeks	7.93 (95% CI: 0.78, 80.68; p=0.08)	0.99 (95% CI: 0.42, 2.37; p=0.985)	NA	4.25 (95% CI: 0.53, 34.31; p=0.175)	5.29 (95% CI: 0.54, 51.57; p=0.152)	5.28 (95% CI: 0.54, 51.47; p=0.152)
Placebo	1.87 (95% CI: 0.68, 5.12; p=0.225)	0.23 (95% CI: 0.03, 1.78; p=0.16)	0.24 (95% CI: 0.03, 1.9; p=0.175)	NA	1.24 (95% CI: 0.5, 3.09; p=0.637)	1.24 (95% CI: 0.5, 3.08; p=0.64)
Secukinumab 300 mg every 2 weeks	1.5 (95% CI: 0.39, 5.83; p=0.558)	0.19 (95% CI: 0.02, 1.73; p=0.14)	0.19 (95% CI: 0.02, 1.85; p=0.152)	0.8 (95% CI: 0.32, 1.99; p=0.637)	NA	1 (95% CI: 0.39, 2.55; p=0.997)
Secukinumab 300 mg every 4 weeks	1.5 (95% CI: 0.39, 5.84; p=0.556)	0.19 (95% CI: 0.02, 1.74; p=0.141)	0.19 (95% CI: 0.02, 1.85; p=0.152)	0.81 (95% CI: 0.32, 2; p=0.64)	1 (95% CI: 0.39, 2.56; p=0.997)	NA

League Table for the Primary Network Analysis on the Occurence of Severe Adverse Events. The upper triangle shows pooled odds ratios for available direct treatment comparisons. The lower triangle contains network estimates of odds ratios for all possible treatment comparisons. The 95% CI is provided in brackets. CI = confidence interval; NA = not applicable.

Table D2

Comparison	Prop	Combined	Direct	Indirect	Diff	p-value
Adalimumab 40 mg every week vs Bimekizumab 320 mg every 2 weeks	0	-2.08	NA	-2.08	NA	NA
Adalimumab 40 mg every week vs Bimekizumab 320 mg every 4 weeks	0	-2.07	NA	-2.07	NA	NA
Adalimumab 40 mg every week vs Placebo	1	-0.62	-0.62	NA	NA	NA
Adalimumab 40 mg every week vs Secukinumab 300 mg every 2 weeks	0	-0.41	NA	-0.41	NA	NA
Adalimumab 40 mg every week vs Secukinumab 300 mg every 4 weeks	0	-0.41	NA	-0.41	NA	NA
Bimekizumab 320 mg every 2 weeks vs Bimekizumab 320 mg every 4 weeks	1	0.01	0.01	NA	NA	NA
Bimekizumab 320 mg every 2 weeks vs Placebo	1	1.45	1.44	NA	NA	NA
Bimekizumab 320 mg every 2 weeks vs Secukinumab 300 mg every 2 weeks	0	1.67	NA	1.67	NA	NA
Bimekizumab 320 mg every 2 weeks vs Secukinumab 300 mg every 4 weeks	0	1.67	NA	1.67	NA	NA
Bimekizumab 320 mg every 4 weeks vs Placebo	1	1.45	1.43	NA	NA	NA
Bimekizumab 320 mg every 4 weeks vs Secukinumab 300 mg every 2 weeks	0	1.67	NA	1.67	NA	NA
Bimekizumab 320 mg every 4 weeks vs Secukinumab 300 mg every 4 weeks	0	1.66	NA	1.66	NA	NA
Secukinumab 300 mg every 2 weeks vs Placebo	0.99	-0.22	-0.19	-2.72	2.53	0.54
Secukinumab 300 mg every 4 weeks vs Placebo	0.99	-0.22	-0.18	-2.76	2.57	0.53
Secukinumab 300 mg every 2 weeks vs Secukinumab 300 mg every 4 weeks	1	0	0	NA	NA	NA

Net Splitting for the Primary Network Analysis on the Occurrence of Severe Adverse Events. Net splitting did not show any inconsistency in the network. Most combined treatment effects were derived by either direct of indirect evidence. Prop = direct evidence proportion used for estimating the treatment effect in the network meta-analysis; Combined = estimated treatment effect in the network meta-analysis; Direct = estimated treatment effect derived from direct evidence; Indirect = estimated treatment effect derived from indirect evidence; Diff = difference between direct and indirect effect estimate; p-value = p-value of test for disagreement between direct and indirect effect estimate; NA = not applicable.

Figure D1

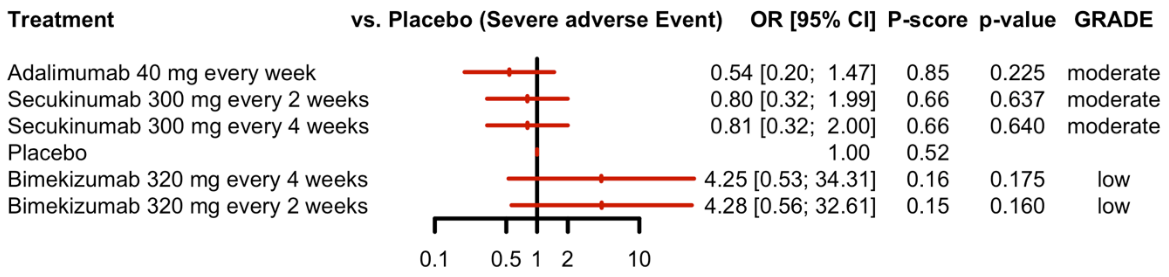

Forest Plot for the Primary Network Analysis on the Occurrence of Severe Adverse Events. The odds ratio for experiencing a severe adverse event is shown for all treatments with placebo as comparator. The treatments are ranked according to P-scores. The certainty of evidence is provided on the right (GRADE). OR = odds ratio; CI = confidence interval; GRADE = GRADE Working Group approach for rating the quality of effect estimates from network meta-analysis.

Figure D2

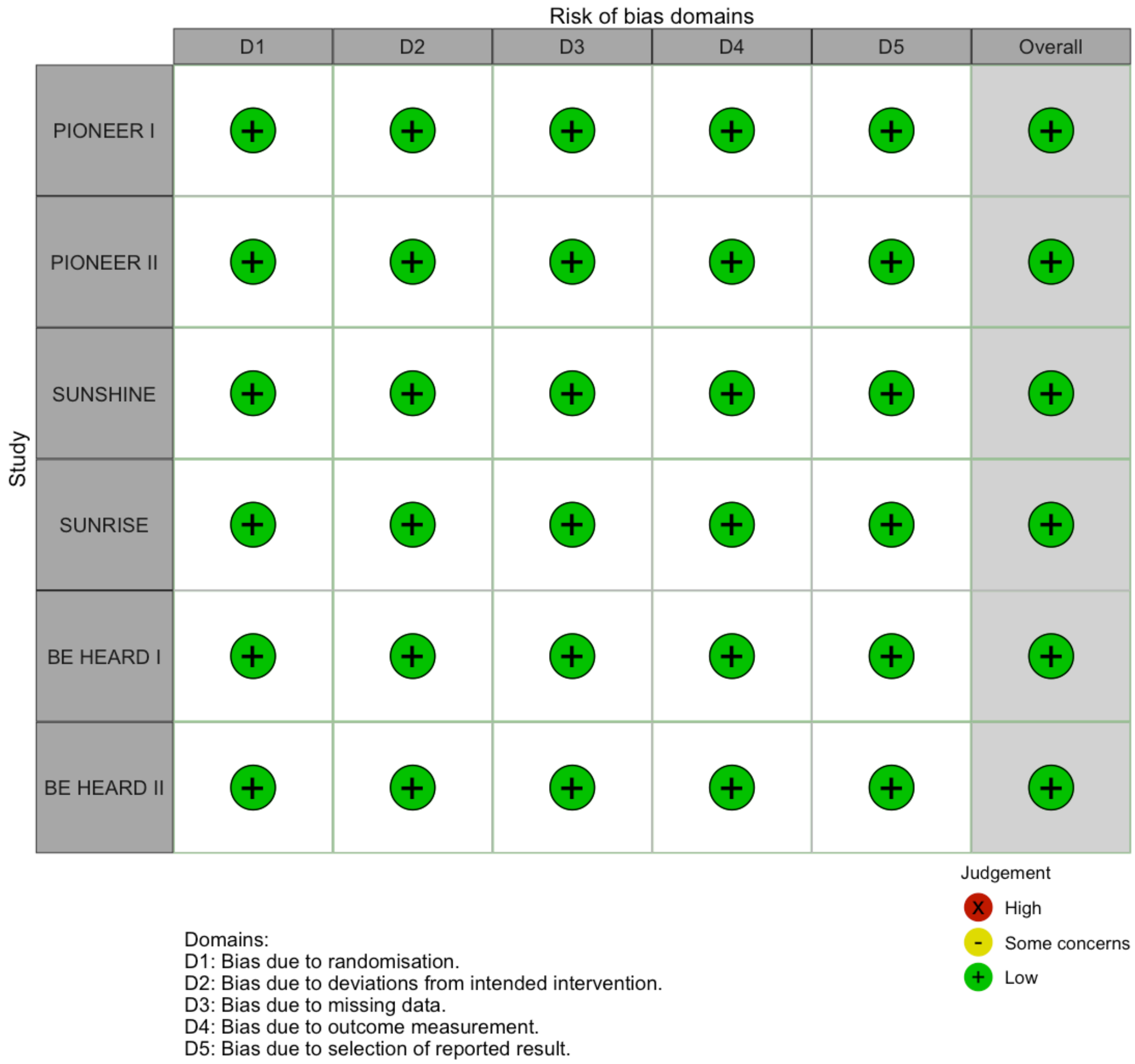

Risk of Bias Assessment for Severe Adverse Events. The judgements for each trial providing data for the network meta-analysis on severe adverse events are shown in this traffic lights plot.

Figure D3

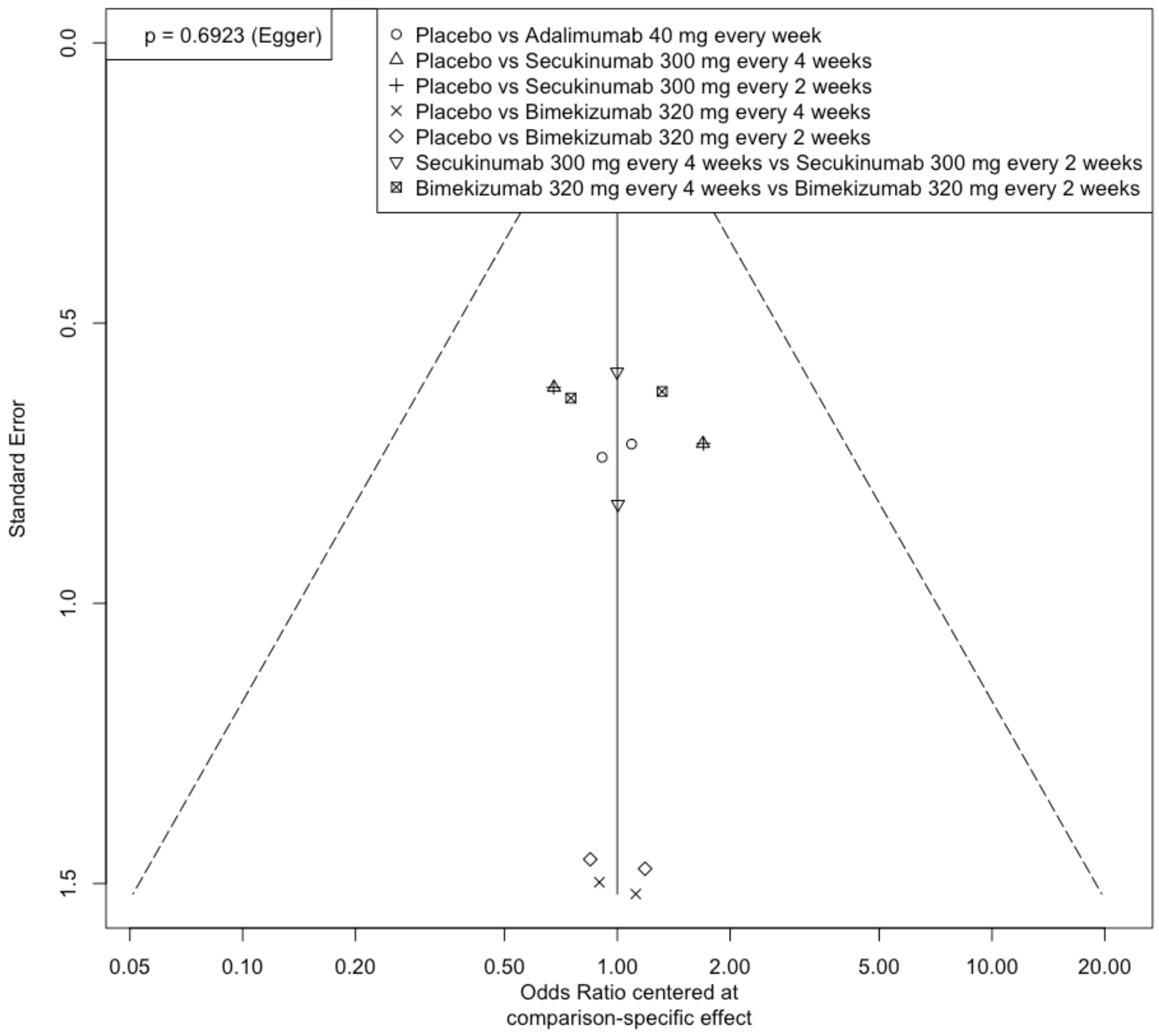

Comparison-Adjusted Funnel Plot for Network Analysis on the Occurrence of Severe Adverse Events. The p-value of Egger's test is shown in the top left-hand corner.

Appendix E

To date, few NMAs focused on systemic treatments of HS. None of these NMAs included data from the recently concluded phase III trial of bimekizumab, and all of these NMAs analyzed trials at different phases, which resulted in a certain degree of heterogeneity in methodology and baseline characteristics among included studies. One NMA, conducted in 2022, included RCTs that tested not only biologics (infliximab, adalimumab, anakinra and bermekimab) but also botulinum toxin, oral tetracycline, and clindamycin (3). The outcome HiSCR50 was not available for all included studies. The results of this NMA showed that bimekizumab had the highest probability for the occurrence of a clinical response (HiSCR50) compared to adalimumab, anakinra and placebo (3). One possible explanation for why Gupta et al. reported higher efficacy of bimekizumab compared with adalimumab, which contrasts with our results, is that their analysis included data from the phase II trial of bimekizumab for moderate-to-severe HS, as phase III data were not available at that time. The higher efficacy observed in their study may be attributed to the nature of phase II trials, which often involve a more controlled and homogeneous patient population and may not fully capture the variability of real-world clinical settings. In contrast, phase III trials are designed to provide a more comprehensive evaluation across diverse patient populations and clinical scenarios, typically resulting in more generalizable but less pronounced efficacy estimates. Another meta-analysis was conducted on nine biologics and three small-molecule inhibitors for the treatment of moderate-to-severe HS, including, among others, adalimumab, anakinra, bimekizumab, etanercept, guselkumab, infliximab and bermekimab (4). No trials on secukinumab and only phase II studies on bimekizumab were included in that study. The meta-analysis revealed that only adalimumab and bimekizumab achieved a significant improvement of symptoms measured by HiSCR50, with adalimumab achieving slightly better results. Finally, one recent NMA published in 2023 included RCTs on biologics (adalimumab, secukinumab and bimekizumab) as well as on small molecules (5). Only phase II data on bimekizumab was included. In terms of the probability for achieving HiSCR50 at 12–16 weeks, adalimumab ranked first, followed by bimekizumab and secukinumab. The biologics and small molecules did not differ in the development of AEs compared to placebo (5).

References

1. Sterne JAC, Savović J, Page MJ, Elbers RG, Blencowe NS, Boutron I, et al. RoB 2: a revised tool for assessing risk of bias in randomised trials. Bmj. 2019;366:l4898.
2. Puhan MA, Schünemann HJ, Murad MH, Li T, Brignardello-Petersen R, Singh JA, et al. A GRADE Working Group approach for rating the quality of treatment effect estimates from network meta-analysis. Bmj. 2014;349:g5630.
3. Gupta AK, Shear NH, Piguet V, Bamimore MA. Efficacy of non-surgical monotherapies for hidradenitis suppurativa: a systematic review and network meta-analyses of randomized trials. J Dermatolog Treat. 2022;33(4):2149-60.
4. Huang CH, Huang IH, Tai CC, Chi CC. Biologics and Small Molecule Inhibitors for Treating Hidradenitis Suppurativa: A Systematic Review and Meta-Analysis. Biomedicines. 2022;10(6).
5. Tsai YC, Hung CY, Tsai TF. Efficacy and Safety of Biologics and Small Molecules for Moderate-to-Severe Hidradenitis Suppurativa: A Systematic Review and Network Meta-Analysis. Pharmaceutics. 2023;15(5).
